# Harmful somatic amino acid substitutions affect key pathways in cancers

**DOI:** 10.1186/s12920-015-0125-x

**Published:** 2015-08-19

**Authors:** Abhishek Niroula, Mauno Vihinen

**Affiliations:** Department of Experimental Medical Science, Lund University, BMC B13, SE-22184, Lund, Sweden

**Keywords:** Cancer genomes, Somatic mutations, Cancer pathways, Cancer relationship

## Abstract

**Background:**

Cancer is characterized by the accumulation of large numbers of genetic variations and alterations of multiple biological phenomena. Cancer genomics has largely focused on the identification of such genetic alterations and the genes containing them, known as ‘cancer genes’. However, the non-functional somatic variations out-number functional variations and remain as a major challenge. Recurrent somatic variations are thought to be cancer drivers but they are present in only a small fraction of patients.

**Methods:**

We performed an extensive analysis of amino acid substitutions (AASs) from 6,861 cancer samples (whole genome or exome sequences) classified into 30 cancer types and performed pathway enrichment analysis. We also studied the overlap between the cancers based on proteins containing harmful AASs and pathways affected by them.

**Results:**

We found that only a fraction of AASs (39.88 %) are harmful even in known cancer genes. In addition, we found that proteins containing harmful AASs in cancers are often centrally located in protein interaction networks. Based on the proteins containing harmful AASs, we identified significantly affected pathways in 28 cancer types and indicate that proteins containing harmful AASs can affect pathways despite the frequency of AASs in them. Our cross-cancer overlap analysis showed that it would be more beneficial to identify affected pathways in cancers rather than individual genes and variations.

**Conclusion:**

Pathways affected by harmful AASs reveal key processes involved in cancer development. Our approach filters out the putative benign AASs thus reducing the list of cancer variations allowing reliable identification of affected pathways. The pathways identified in individual cancer and overlap between cancer types open avenues for further experimental research and for developing targeted therapies and interventions.

**Electronic supplementary material:**

The online version of this article (doi:10.1186/s12920-015-0125-x) contains supplementary material, which is available to authorized users.

## Background

Cancer is characterized by the accumulation of large numbers of genetic variations and alterations of multiple biological phenomena [1, 2]. These alterations contribute directly or indirectly to increased ratio of cell birth to cell death [3]. During recent years, cancer genomics has largely focused on the identification of such genetic alterations and the genes containing them, known as ‘cancer genes’. Variations that confer growth advantage and are positively selected during cancer development are known as drivers and other variations carried along during cancer progression are called for passengers [4]. Recurrent somatic variations are thought to be drivers but they are present in only a small fraction of patients. On the other hand, previous studies showed that less frequent variations can have similar effects as recurrent variations [5, 6].

Large amounts of cancer genomic data are available by joint efforts of various genomic projects. These include the Cancer Genome Project (CGP) (https://www.sanger.ac.uk/research/projects/cancergenome/), The Cancer Genome Atlas (TCGA) (http://cancergenome.nih.gov/) and International Cancer Genome Consortium (ICGC) [7]. Massive datasets provide unprecedented possibilities for data analysis. Various approaches have already been taken to understand the mechanisms of tumorigenesis [8]. However, the vast majority of non-functional somatic variations remain the major challenge [9].

Here, we exploited the impacts of somatic amino acid substitutions (AASs) to prioritize relevant variations in cancers and identified pathways affected by them. We utilized PON-P2 [10], a machine learning-based tool to identify harmful AASs. It classifies the AASs into three categories: pathogenic, neutral and unknown. Those AASs that are predicted with confidence level 0.95 are classified either as pathogenic or neutral and the remaining as unknown. Here, all AASs that were classified as pathogenic by PON-P2 were considered to be likely harmful. As cancer is a multigenic disease, single variants cannot be called pathogenic and therefore, we name them as ‘harmful’. PON-P2 does not predict mechanisms of AASs, instead it identifies deviations from normal amino acids in the sequence positions. This means that harmful AASs can be of either loss or gain of function type.

In our analysis, we found that only a small fraction of AASs are harmful even in known cancer proteins. Proteins containing harmful AASs in cancer are often centrally located in protein interaction networks and they affect key pathways. Even proteins with low AAS frequency can affect key cancer pathways. We performed cross-cancer comparison based on prioritized proteins and affected pathways. Our analysis showed that cancers have higher similarities at pathway level than at protein level. Hence, it would be more beneficial to identify affected pathways in cancers than individual genes/proteins and variations.

## Results

**Fig. 1:**
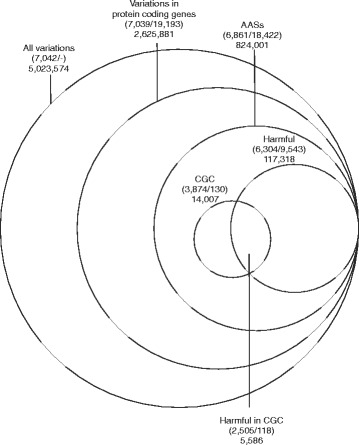
Variations in cancer. Venn diagram illustrates the numbers of samples, genes and variations at different levels during data filtration. The figures in the brackets are numbers of samples and numbers of genes, respectively. The values outside brackets are numbers of variations

We obtained 5,023,574 somatic variations in 7,042 cancer samples in 30 cancer types [11]. We mapped the variations to human reference sequence and identified 824,336 single nucleotide variations (SNVs) leading to AASs in altogether 6,861 samples (Fig. 1). The numbers of variations leading to synonymous alterations (308,896) and introducing stop codons in mRNA (63,866) are much smaller compared to the number of AASs. The ratio of non-synonymous to synonymous mRNA variations varies among cancer types. It ranges from 1.8 in melanoma to 6.7 in lung small cell cancer while the overall ratio is 2.7. Even minor genetic changes can provide advantage for cancer cells. However, as practically all cancers contain harmful AASs, it is highly relevant to study proteins containing them and their biological processes. We utilized PON-P2 (http://structure.bmc.lu.se/PON-P2/) [10], a highly reliable tool, for identification of harmful AASs. Here, all AASs classified as pathogenic by PON-P2 are considered to be harmful. In total, 14.24 % of AASs were predicted as harmful in 91.88 % of the samples. AASs are common in cancers except in pilocytic astrocytoma and liver cancer, which contained AASs in only 32.67 % and 55.68 % of samples, respectively (Additional file 1: Table S1). The frequencies of AASs vary between and within the cancer classes (Additional file 2: Figure S1). Several factors including the age of patient at the time of sequencing, exposure to mutagens, microsatellite instability, etc. contribute to the frequency of variations.

### Proportions of harmful variations are higher in cancer genes

Cancer Gene Census (CGC) [12] catalogues 138 genes in which somatic variations leading to AASs are causally implicated in cancer. Only 56.46 % of the samples contained AASs in proteins translated from altogether 130 CGC genes (Additional file 1: Table S1) and 36.51 % of the samples contained harmful variations in totally 118 CGC genes (Fig. 1). The proportion of harmful variations is higher in CGC genes (39.88 %) compared to the whole dataset (14.24 %). In total, 4.76 % of the harmful variations were present in CGC genes.

Catalogue of Somatic Mutations in Cancer (COSMIC) [13] release 68 contains 1,646,844 (1,293,087 unique) somatic variations. Using PON-P2, 14.71 % of the unique variations leading to AASs were predicted to be harmful in 9,140 genes in 43.89 % of the samples (available at http://structure.bmc.lu.se/PON-P2/cancer30.html/). In total, 96.55 % of the samples contain 15,176 AASs in the translated protein sequences of 124 CGC genes, 40.98 % of them are predicted as harmful (Additional file 1: Table S2). These results confirm that variations in the cancer genes are more often harmful than variations on average, however, the far majority of the variations are benign or have only minor effect even in the cancer genes. We analyzed the most frequent variants present in more than 25 samples in COSMIC, altogether 327 AASs in 61 proteins. These frequent AASs show high predicted probability of harmfulness (mean = 0.76 and median = 0.83) (Additional file 3: Figure. S2a). There are large numbers of less frequent AASs with similar probabilities of harmfulness. Thus, frequent variations are often harmful, but less frequent variations can be equally harmful.

### Evaluation of PON-P2 on cancer variant datasets

The performance of PON-P2 has been extensively validated and compared to different tools [10]. To assess the performance of PON-P2 for cancer variants, we used three somatic variation datasets. We collected the pathogenic somatic variations from ClinVar [14], Database of Curated Mutations (DoCM) (http://docm.genome.wustl.edu/) and TP53 mutation database [15]. In total, there were 1,058 AASs in 82 proteins. The distribution of the probability of harmfulness is similar for all three datasets and the probabilities are concentrated near 1 (Additional file 3: Figure S2b). In total, 733 (69.3 %) AASs were predicted as harmful, 4 as benign and the remaining 321 were unclassified. To estimate the false positive rate of PON-P2, we took the AASs that were annotated as not showing significant difference in protein activity from the TP53 mutation database. Among 454 AASs, only 87 were predicted as harmful thus showing a low false positive rate (19.2 %). However, this number may be an overestimate as there are results only for one single protein and there is a possibility of random effects. True test would require a much larger dataset for AASs in several proteins. In the PON-P2 benchmark test data for 1,605 benign AASs the corresponding false positive rate is only 8.97 %. As the distributions of predicted probabilities were similar for the cancer datasets, we investigated the overlap between them. There is very little overlap between them, however the individual datasets overlap to some extent with the frequent AASs in COSMIC (Additional file 3: Figure S2c-d).

### Landscape of somatic AASs

The landscape for variations leading to harmful AASs was compared to that for the entire dataset. All possible base substitutions are represented by six classes of substitutions, C > T, C > A, C > G, T > C, T > A and T > G. C > T substitution is the most prevalent base alteration in most cancers and even more prominent among harmful substitutions leading to amino acid alterations (Additional file 2: Figure. S1). There are differences between cancer types: C > A substitution is common in the three types of lung cancer and neuroblastoma while C > G substitutions are enriched in the cancers of bladder and cervix. The landscape was investigated also based on the base substitutions and their immediate 5’ and 3’ nucleotides. The majority of the variations are C > T substitutions in CpG and TpC dinucleotides. In some of the cancers, C > G and C > A substitutions are enriched in TpC sites (Additional file 4: Figure S3). Among the harmful variations, the C > G substitutions are less frequent in most of the cancers. C > A substitutions remain prevalent in the three types of lung cancer and T > C substitutions in the liver cancer (Additional file 4: Figure S4).

We studied the patterns of AASs in each cancer type. Arginine is the most frequently substituted residue in both datasets (AASs and harmful AASs) while the substitutions from alanine and glutamate are less frequent among harmful AASs (Additional file 5: Figures S5 and S6). The most common harmful substitutions are R > H, R > W, R > C and E > K. The high frequency of arginine may be explained by its six codons, four of which have CpG dinucleotide, a well-known mutation hotspot [16]. On the other hand, glutamic acid is coded by only two codons and neither of them contains CpG. There are cancer type specific differences in the AAS distribution. For example in the lung cancers G > V substitutions are prevalent and in liver and thyroid cancers Y > C substitutions are prevalent (Additional file 5: Figure S6).

**Fig. 2:**
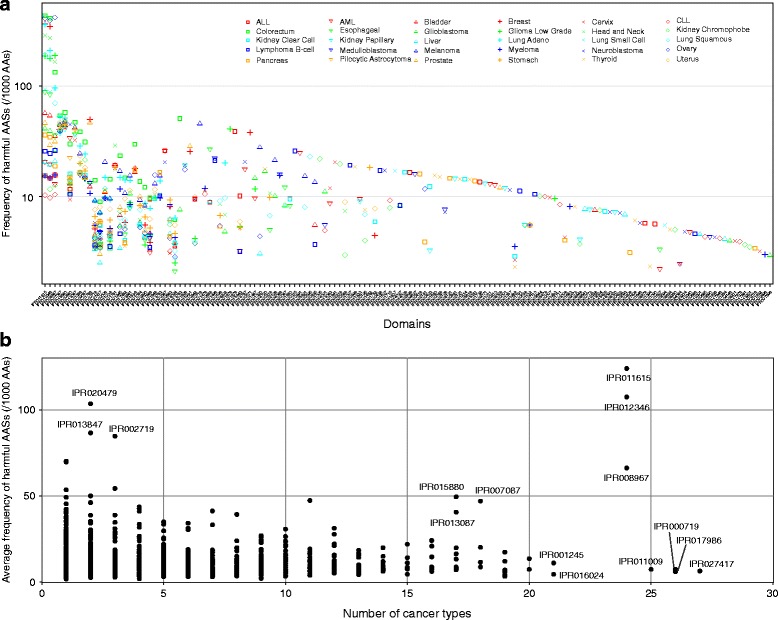
Distribution of harmful AASs in protein domains. **a** Frequency of harmful AASs in InterPro domains. The top 20 domains containing the highest numbers of harmful AASs in each cancer typeare shown. **b** Prevalence of AASs in domains in multiple cancers and frequency of harmful AAS

We studied also the distribution of AASs in protein domains. The p53 DNA binding domain has the highest AAS frequency in multiple cancers (Fig. 2a and Additional file 1: Table S3). Zinc finger domains have the second highest frequency among AASs and harmful AASs. We compared the protein domains with the most frequent AASs and harmful AASs in each cancer. We selected top 20 domains containing the highest AAS frequencies in each cancer. In total, 93 and 147 domains were selected for all AASs and harmful AASs, respectively (Fig. 2a, Additional file 6: Figure S7a). Among them, 70 domains overlap between the two sets. We compared also the frequency of AASs in domains in all the cancers together. The p53 DNA binding domain contained the highest frequency of harmful AASs in altogether 24 cancer types (Fig. 2b). In the data for all AASs, some domains contain AASs in more than 24 cancer types, however with a low frequency (Additional file 6: Figure. S7b). Epidermal growth factor receptor (EGFR) illustrates the distribution of AASs in protein domains. There are 233 AASs (202 unique) in 21 cancer types. 73.3 % (148) of the unique AASs were predicted as harmful altogether in 18 cancer types. The AASs are scattered along the protein chain with slight enrichment in the kinase domain (Additional file 7: Figure. S8) while the harmful AASs are concentrated in the kinase domain. 56.6 % (86) of the harmful AASs appear in the kinase domain that represents one-fourth of the entire protein sequence. The harmful AASs appear frequently in secondary structural elements and likely affect the protein fold (Additional file 7: Figure. S8c). Overall, the benign AASs are located mainly on surface loops or in the termini of the α- and β-structures.

### Prioritizing most relevant proteins

Next we identified proteins containing the highest numbers of harmful AASs in each cancer. As tumor cells multiply rapidly, the number of random variations also increases rapidly however many proteins containing harmful AASs may not have any implications in cancer. Therefore, we eliminated proteins that did not contain harmful AASs in at least two samples in each cancer type. Then we selected proteins that contained harmful AASs in the largest numbers of samples. In addition, proteins containing at least one harmful AAS in at least 2 % of the samples in a cancer type were selected. The latter step was introduced to include proteins with frequent harmful AASs even when the number of affected samples was less than the threshold. The number of selected proteins varied from 2 to 251 depending on cancer type (Additional file 1: Table S4). Several of the genes corresponding to the selected proteins are from CGC but there are numerous novel candidate genes (Additional file 8: Figure. S9).

Since some of the selected proteins have very long sequences (TTN, SYNE1, RYR2, RYR3 etc.), we normalized the frequencies of harmful AASs by the lengths of the reference protein sequences. Proteins with higher normalized frequencies of harmful AASs (Additional file 1: Table S4) are likely implicated in cancer. Further studies could be prioritized based on the frequencies of the variations causing harmful AASs in these selected proteins.

### Gene Ontology and pathway enrichment

About half of the cancers have only a small number of selected proteins (<20) (Additional file 1: Table S4). In these cancers, genes corresponding to proteins containing at least one harmful AAS were further analyzed. For the other cancer types, we used the genes corresponding to selected proteins and performed Gene Ontology (GO) and pathway enrichment analysis in each cancer type. GO terms associated with biological processes like cell differentiation, cell death, cell cycle and more specific terms are significantly enriched in many cancer types (Additional file 1: Table S5). Significantly affected pathways include cell cycle, apoptosis, signaling by NOTCH, PI3K, mTOR, MAPK, Wnt, EGFR, PDGF, and others (Fig. 3, Additional file 1: Table S6 and Additional file 9: Figures S10-S37). Examples in head and neck cancer (HNC) and acute lymphocytic leukemia (ALL) are discussed to highlight the observations.

**Fig. 3:**
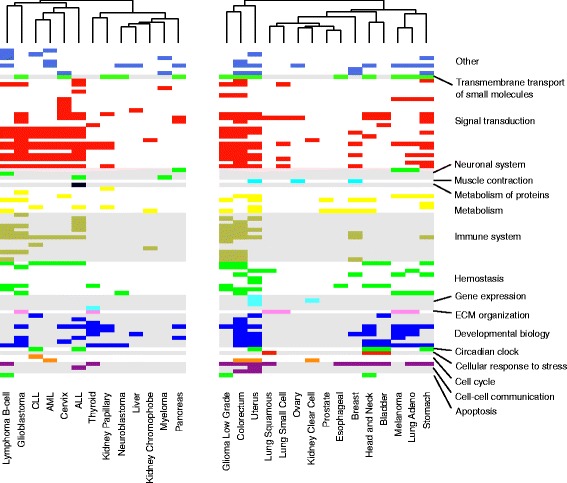
Significantly enriched pathways in cancers. Each row represents a pathway. They are clustered based on the tree structure in Reactome pathway database. Pathway enrichment was performed using all proteins containing harmful AASs (*left*) or using selected proteins (*right*). The cancer types are clustered by complete linkage hierarchical clustering method. Medulloblastoma and pilocytic astrocytoma are excluded as no pathways were significantly enriched

### Head and neck cancer (HNC)

**Fig. 4:**
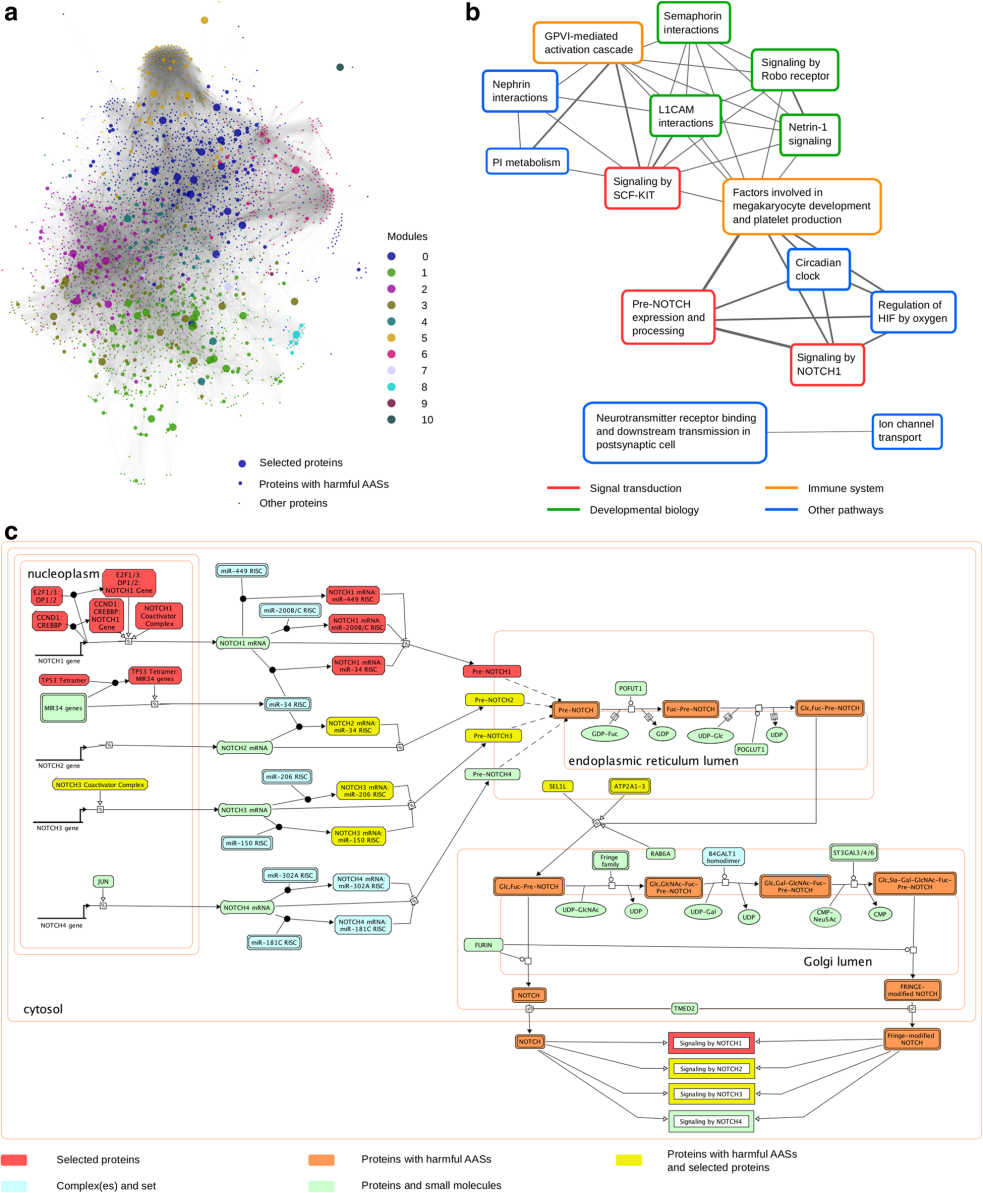
Networks of proteins and pathways affected in HNC. **a** The selected proteins and their first neighbors in Reactome functional interaction network are highly connected. The nodes were clustered into modules (*indicated by colors*) using ReactomeFI plugin in cytoscape. **b** Statistically significantly enriched pathways (*Reactome pathway database*) affected in HNC. Nodes represent pathways and edges represent common proteins having roles in the connected pathways. The edge line thickness represents the number of common proteins in the two pathways. Only the selected proteins in HNC are included. **c** Pre-NOTCH expression and processing pathway from Reactome pathway database. This is the most frequently affected pathway in HNC samples

In HNC, we selected 56 proteins that contain at least one harmful AAS totaling 62.11 % of the samples. The corresponding genes for 10 of these proteins (*TP53*, *EP300*, *EGFR*, *CREBBP*, *NFE2L2*, *FBXW7*, *NOTCH1*, *PIK3CA*, *RAC1* and *STAG2*) are catalogued in CGC. In addition, *FAT1*, *SYNE1* and *TP63* have been reported as significantly affected genes in HNC [17]. Our study revealed 43 additional candidate genes (Additional file 1: Table S4). Enrichment analysis of GO terms pinpointed biological processes including cell differentiation and multicellular organization (False Discovery Rate (FDR) < 0.001) (Additional file 1: Table S5). In the functional interaction network extracted from ReactomeFI, the selected proteins are highly connected (Fig. 4a). In a network, the degree of a node is the number of direct links of the node in the network. The nodes for selected proteins have higher average degree (95.2) compared to the nodes representing other proteins containing harmful AASs (49.0) and the overall degree of the nodes in the complete network (32.0). The proteins frequently containing harmful AASs are thus centrally located in the functional interaction networks. The selected proteins are distributed in several functional modules (Fig. 4a). Pathway enrichment analysis of the modules shows distinct pathways enriched in the modules. Pathways involved in transcription and its regulation are enriched in module 0. Cell surface interaction and muscle contraction pathways are enriched in module 1. Signaling pathways are enriched in modules 2, 3 and 4. NOTCH signaling, DNA replication and DNA replication are enriched in modules 5 and 7. Pathways of cell division are enriched in module 6. Hence, several pathways are affected by the prioritized proteins in HNC.

We identified the significantly enriched pathways (FDR <0.05) (Additional file 1: Table S6). To unveil overlap between pathways, we generated a network of significant pathways (Fig. 4b). Factors involved in megakaryocyte development and platelet production have the highest degree i.e. proteins involved in this pathway are shared with many other pathways. NOTCH signaling pathway is the most frequently affected pathway in HNC. It includes NOTCH1-NOTCH4 signaling and pre-NOTCH expression and processing pathways. These pathways are affected in altogether 78 samples (20.53 % of all the HNC samples) with harmful AASs in at least one of the proteins corresponding to the 5 genes (*EP300*, *CREBBP*, *FBXW7*, *TP53*, and *NOTCH1*) all of which are in the CGC. When we consider also variations that lead to substitution by stop codon, insertions and deletions in these 5 genes, the number of affected samples increases to 160 (42.1 %). These additional variation types are very likely harmful due to large alterations to genes and coded proteins. Other proteins involved in the pathway are the products of *NOTCH2*, *NOTCH3*, *SEL1L* and *ATP2A2*, all of which contain variations leading to harmful AASs in more than one sample (Fig. 4c). These proteins contained harmful AASs in 11 additional samples. Similar results are obtained in other cancer types where cancer related central pathways are affected by proteins with harmful AASs at different frequencies (Additional file 9: Figures S10-S37). Thus, harmful AASs in a cancer type impair proteins involved in different functions within certain pathway. Previously, idiosyncratic variations were found to have similar effects as recurrent variations [5, 6]. Hence, it is essential to investigate also variations occurring at low frequency and explore the pathways affected by them.

NOTCH signaling pathway (Fig. 4c) is highly conserved in most multicellular organisms and regulates cell differentiation, proliferation, and cell-fate determination. It has been reported to be affected in various cancers including HNC [17–19] and is emerging as a new therapeutic target. Another significantly enriched pathway is SCF-KIT pathway, which is affected in several cancer types including HNC. The pathway contains stem cell factor (SCF) and its receptor KIT. SCF homodimer binds to KIT activating the tyrosine kinase domain. Then, KIT stimulation activates several signaling pathways including RAF/MAP kinase, AKT and JAK/STAT pathways.

### Acute lymphocytic leukemia (ALL)

In ALL, only 2 proteins (encoded by *PHF6* and *NOTCH1*) were selected, therefore we included all genes containing one or more variations leading to harmful AASs. GO enrichment analysis indicates that biological processes including cell differentiation, cell proliferation, and developmental processes are significantly enriched (FDR < 0.01) (Additional file 1: Table S5). Similar to HNC, proteins containing harmful AASs in more than one sample have higher connectivity with an average degree of 196.6 compared to other proteins containing harmful AASs (110.7) and the overall degree of the nodes in the functional interaction network (32.0) (Additional file 10: Figure S38). Pathway enrichment analysis identified significant pathways (FDR <0.05) (Additional file 10: Figure S39). There are 53 proteins with harmful AASs involved in significantly enriched pathways, 16 of which have their corresponding genes in CGC. In the network of pathways, immune system and signaling pathways are highly connected (Additional file 10: Figure S39). Factors involved in megakaryocyte development are affected in 13 samples (13.3 %) containing harmful AASs in proteins corresponding to *GATA2, GATA3, DOCK2, MYB, CREBBP, TP53* and *EP300* genes. Including the insertions, deletions and nonsense substitutions, these 7 proteins contain AASs in 23 samples (23.5 %). Pre-NOTCH expression and processing is also affected in 13 samples. Other significantly affected pathways include transcription regulation of white adipocytes and SCF-KIT signaling pathway.

### Cancer network

**Fig. 5:**
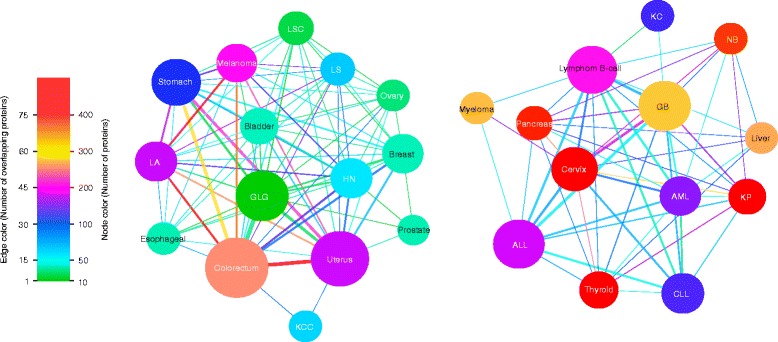
Cancer network. Nodes represent cancer types and edges indicate shared significantly enriched pathways between them. Node size represents the number of significant pathways affected in the cancer type and node color represents the number of proteins. Edge line width represents the number of common pathways between the cancers and edge color represents the number of common proteins between the cancers. GB, Glioblastoma; GLG, Glioma low grade; HN, Head and neck; KC, Kidney chromophobe; KCC, Kidney clear cell; KP, Kidney papillary; LA, Lung adeno; LS, Lung squamous; LSC, Lung small cell; NB, Neuroblastoma

Large scale genomic studies have revealed the heterogeneous nature of cancers. Variation patterns are diverse even in tumors originating from the same tissue or organ [11, 20] while similar patterns of genomic alterations are observed in cancers from different tissues of origin [21]. We evaluated the similarities between cancer types based on the affected pathways. We generated a network for cancers which have more than 20 selected proteins and another network for the remaining cancers (Fig. 5). The nodes are highly connected to each other in both networks indicating that cancers share several pathways that contain harmful AASs even when they share fewer proteins. Variations can affect pathways at any step and therefore pathways are more relevant for cancer than individual genes and proteins. In Mendelian diseases, several examples are known of related diseases originating due to variations in proteins in the same signaling or metabolic pathways [22, 23]. Also in cancers, it may not be that relevant which protein in a pathway is affected since they all would impair the function of the system and contribute to cancer.

## Discussion

We analyzed somatic AASs in 6,861 cancer samples (whole genome or exome sequences) classified into 30 cancer types. Several methods including MutSigCV [20], MuSic [24], InVEx [25], Oncodrive [26, 27] and HotNet2 [28] have been developed to analyze the cancer genomes and identify cancer variants, genes, networks and pathways. Although some highly relevant cancer genes have been identified based on the assumption that genes with higher variation frequency than the background mutation rate are putative drivers, large numbers of tumors do not have any variations in these genes. Several variations leading to AASs in well-known driver genes do not have functional impact, for example in *TP53* [6]. Thus, the numbers of tumors with harmful variations in driver genes is even lower than previously presented (Tables S1 and S2). Here, we took a novel approach to identify harmful somatic AASs and to reveal pathways affected by them. Due to lack of benchmark datasets, it is not possible to compare the performance of PON-P2 to the methods based on substitution frequencies. We evaluated the applicability of PON-P2 on three cancer variation datasets. The validated cancer variants obtained high predicted probabilities of harmfulness (Additional file 3: Figure. S2). PON-P2 reliably identified 69.3 % of AASs as harmful and 0.4 % as benign. The remaining AASs were predicted as unknown.

Our study revealed that many variations in known cancer genes are highly likely benign although the numbers of harmful variations in these proteins are higher than on average in proteins (Additional file 1: Tables S1 and S2). The relevance of harmfulness of AASs in cancer is further evidenced by our analysis of AASs in COSMIC. Frequent somatic AASs are highly likely harmful with high predicted probabilities (Additional file 3: Figure S2). The distribution of the probabilities of harmfulness for frequent AASs in COSMIC is very similar to those for the three additional somatic variation datasets. Also, large numbers of less frequent AASs are harmful many of which may have been introduced by random chance. The harmful AASs appear in proteins that have higher degree of connectivity in the functional interaction networks. The proteins that contain harmful AASs frequently are even more connected which is similar to cancer proteins in a previous study [29] (Fig. 4a and Additional file 10: Figure S38). Pathway enrichment analysis revealed proteins with varying numbers of harmful AASs involved in key cancer-related pathways (Additional file 9: Figures S10-S37) confirming that cancer is an outcome of large numbers of accumulated harmful effects in a number of proteins and pathways. In addition, although cancer types have common affected pathways (Fig. 5), there are different pathways specific for individual cancer types (Additional file 1: Table S6 and Additional file 9: Figures S10-S37). Only a fraction of AASs in any protein are highly deleterious. Recent analysis of predicted harmful AASs in the kinase domain of Bruton tyrosine kinase revealed that 67 % of the single nucleotide change caused AASs are harmful [30]. This number is higher than in most previous studies due to the importance of the kinase domain ([30] and the references therein).

To detect proteins most relevant in cancers, we prioritized approximately the top 5 % proteins in each cancer type based on the number of samples containing harmful AASs in them. Some of the prioritized proteins have been previously implicated in cancers while others are novel candidate proteins (Additional file 8: Figure S9). The numbers of the affected proteins vary cancer-wise. The possible reasons are i) there are other types of (genetic) aberrations responsible for cancer development, and/or ii) the cancers may have subtypes such as in breast cancer. As there are large numbers of proteins containing AASs, reliable methods are needed to prioritize the most relevant affected proteins. By doing this with PON-P2, we identified pathways relevant for cancers. We limited our analysis to harmful AASs that were reliably predicted by PON-P2. AASs that were predicted as unknown may contain harmful variations, however they are excluded as the predictions are not reliable for them. Other types of variations were not analyzed as there are not highly reliable predictors for them. Inclusion of other types of genetic variations would further increase the numbers of samples containing harmful variations. Therefore, the presented results and pathways represent the lower boundary of harmful variations. Examples of HNC and ALL revealed relevant candidate genes and key pathways that are involved also in many other cancer types (Additional file 9: Figures S10-S37 and Additional file 1: Table S6). This clearly shows that analysis of cancer genomes in pathway context provides more and richer information than in gene/protein context. Hence, our results suggest that studies of cancer variations should be performed at pathway level based on the effects of variations and would further be supported by additional multi-platform data for example gene expression, copy number variations, miRNA expression, methylation, etc. Our findings provide novel targets for experimental cancer research for understanding processes involved in cancers and for identifying novel targets for therapies.

## Conclusion

In this study, we exploited the impacts of AASs to filter the putative functionally benign variations in cancer genomes. We identified the likely harmful AASs in cancers. Only a small fraction of the AASs are harmful even in well-known cancer proteins. Analyzing the most frequent AASs in the COSMIC database and three other somatic variation datasets, we found that the recurrent AASs are highly likely harmful. However, not all AASs in proteins containing recurrent variations are harmful. We identified the pathways affected by harmful AASs in 28 cancer types. Even proteins with low AAS frequency can affect key pathways relevant for cancer. Therefore, it is essential to identify pathways in cancers instead of proteins/genes. This is further evidenced by the high similarities between cancer types at pathway level rather than at protein level.

## Methods

### Somatic variation data

We retrieved somatic variation data from 7,042 cancer samples (whole genome or exome sequences) classified into 30 cancer types from ftp://ftp.sanger.ac.uk/pub/cancer/AlexandrovEtAl/. Variations marked as filtered and used for signature analysis [11] were retrieved. We mapped the variations to human reference sequence (Ensembl release 69) (http://ensembl.org/) and obtained the AASs in proteins encoding the longest transcripts for each gene.

We also obtained the complete somatic variation data in the Catalogue of Somatic Mutations in Cancer (COSMIC) release 68. We mapped the variations to the human reference sequence (Ensembl release 69) and obtained the AASs in proteins encoding the longest transcript for each gene.

Three datasets were used for assessing the performance of PON-P2 for cancer variants. We obtained somatic variations leading to AASs from ClinVar database [14]. We obtained 65 AASs for which the clinical significance was annotated as pathogenic. 387 disease related AASs were from the Database of Curated Mutations (DoCM) (http://docm.genome.wustl.edu/). The variations in the database are individually curated for clinical and/or functional evidence and all of them are associated with cancer. We obtained 634 AASs that lead to loss of protein activity and 454 AASs that do not show significant loss in protein activity from curated TP53 database (tumours only) [15]. The AASs leading to inactive protein are considered to be harmful and those that do not change the protein activity as benign. All these datasets are freely available in VariBench database (http://structure.bmc.lu.se/VariBench/cancer.php) [31].

### Harmful amino acid substitutions

We identified harmful AASs using PON-P2 (http://structure.bmc.lu.se/PON-P2), a machine learning-based tool [10]. PON-P2 estimates the probability of harmfulness by using 200 predictors. The probability of harmfulness ranges from 0 (likely benign) to 1 (likely harmful). It classifies the confident predictions into harmful and neutral categories with high accuracy. The remaining AASs are called unclassified variants. AASs predicted as harmful were used for the analysis. Predictions for all the AASs analysed in this study including the COSMIC dataset are freely available at http://structure.bmc.lu.se/PON-P2/cancer30.html/.

### Cancer Gene Census

Cancer Gene Census (CGC) lists genes which are causally implicated in cancer [12]. CGC (downloaded February 2014) contained 522 genes in which somatic and/or germline variations are implicated in cancer. Genes that were not reported to have somatic variations leading to AASs were eliminated.

### Lego plots

Lego plots were generated for nucleotide substitutions and AASs in each cancer type using ROOT data analysis framework [32]. For each cancer, two plots were generated, one for all the SNVs leading to AASs and another for SNVs leading to harmful AASs. All possible substitutions are represented by six classes of substitutions, C > T, C > A, C > G, T > C, T > A and T > G (represented by the pyrimidines at the reference nucleotides) and the immediate 5’ and 3’ nucleotides were also considered. The variations in splice sites were excluded for nucleotide substitutions. For AASs, lego plots were plotted for all AASs that are possible by single nucleotide substitutions.

### Protein domains

Annotations for domains were downloaded from InterPro BioMart (http://www.ebi.ac.uk/interpro/biomart/martview/). All the domains were mapped to reference amino acid sequences and AASs in the regions of each domain were identified. To balance the number of AASs and lengths of domains, we normalized the total number of AASs in the region of a domain by the cumulative length of the amino acids in the domains. The cumulative length of the domain is the sum of the lengths of domains containing AASs. Domains that contained more than one AAS were included to the analysis. Protein structures of extracellular domain and DNA binding domain in EGFR protein were obtained from protein data bank (PDB) and visualized using UCSF Chimera visualization tool [33].

### Prioritization of proteins containing harmful AASs

The proteins were prioritized based on the numbers of samples containing harmful AASs in the proteins. Firstly, all harmful AASs in each protein were identified. For each protein, the number of samples containing at least one harmful AAS was counted. Proteins that did not contain harmful AASs in at least two samples were eliminated. The remaining proteins were sorted based on the number of samples. To select approximately top 5 % of the sorted proteins, we set a threshold at 95th percentile for the number of samples in which each protein was affected. Proteins containing harmful AASs in higher number of samples than the threshold were prioritized. To include the recurrent harmful AASs, we also selected those proteins that have at least one AAS in more than 2 % of the samples. This was done only when there were more than 100 samples containing harmful AASs.

### Functional interaction network

Pathway-based protein functional interaction network for proteins coding for human genes was obtained by using the ReactomeFI [34] plugin in cytoscape [35]. The latest version (2013) of the functional interaction network was used where there were 10,706 nodes and 171,449 edges. To reduce the computation time, we reduced the size of the network by removing all nodes except for selected proteins, proteins with harmful AASs and their first neighbours in the cancer type.

Degree of a node is the number of edges by which it is connected to other nodes. We computed the average degree of nodes by using the following equation

$$ {D}_n=\frac{2\times {E}_n+{E}_{other}}{n}, $$

where, n is the number of nodes, D_n_ is the average degree of n nodes, E_n_ is the number of edges connecting any two nodes among n nodes and E_other_ is the number of edges that connect n nodes to other nodes in the network. As the edges connecting two of the n nodes contribute to the degree of both nodes, we multiplied the number of such edges by 2. The overall average degree was computed for the network extracted from ReactomeFI before eliminating any nodes. For overall network, E_other_ is 0 as it is computed for all the nodes in the network.

The proteins in the network were clustered into different modules using the clustering function in the ReactomeFI plugin. Among many significantly enriched pathways, one or two previously reported cancer related pathways were selected for each cancer type. The selected proteins and other proteins containing harmful AASs in the specific cancer types that are involved in the pathways were highlighted manually.

### Enrichment analysis of GO terms and pathways

The Gene Ontology (GO) terms associated with proteins coding for all human genes (GRCh37) were extracted using Ensembl BioMart. GO term enrichment analysis was performed by using the topGO [36] bioconductor package in R. Based on the numbers of selected proteins, the cancers were categorized into two groups. Cancers in which the numbers of selected proteins are below 20 were grouped together. Enrichment analysis of GO terms was performed for all the genes containing at least one harmful variation and GO terms were considered significant at FDR < 0.01. Genes corresponding to selected proteins were used for the remaining cancer types and the GO terms were considered significant if FDR < 0.001. Pathway enrichment analysis was performed by using ReactomeFI plugin in cytoscape. Significantly enriched pathways (FDR < 0.05) were selected.

## Availability of supporting data

All supporting data are included as additional files or kept in publicly available repositories. The somatic variation data used in this article is publicly available at ftp://ftp.sanger.ac.uk/pub/cancer/AlexandrovEtAl/. The validated cancer variation datasets used in this article are freely available in the VariBench database (http://structure.bmc.lu.se/VariBench/cancer.php). Predictions for all AASs supporting the results of this article are freely available at http://structure.bmc.lu.se/PON-P2/cancer30.html/.

## Additional files

Additional file 1:
**This file contains Tables S1 to S9.** Microsoft Excel Workbook containing 6 worksheets. **Table S1**: Variations in each cancer. The numbers of AASs are based on the mapping with the reference sequence (Ensembl release 69) and harmful AASs were predicted by PON-P2. The variations are mapped to proteins translated from longest transcript of CGC genes in COSMIC v68. **Table S2**: Variations in COSMIC database. Numbers of samples, genes/proteins and variations in COSMIC database. **Table S3**: Frequency of harmful AASs in domains. Top 20 domains containing the most number of harmful AASs in each cancer class are shown. The frequency of harmful AASs is normalized by the cumulative length of the amino acid sequence in the domain regions. **Table S4**: Genes corresponding to selected proteins in each cancer. The frequencies of harmful AASs and the numbers of samples containing harmful AASs in the selected proteins are included. **Table S5**: Top GO terms significantly enriched in each cancer. FDR threshold 0.01 was used for cancers in which all the proteins containing harmful AASs were used and FDR threshold 0.001 was used for other cancer types. **Table S6**: Significantly enriched pathways in each cancer. The pathways enriched with FDR lower than 0.5 were considered significant. The pathways are taken from Reactome pathway database. (PDF 268 kb) Additional file 2:
**This file contains supplementary Figure S1.** Frequency of variations leading to AASs in each sample. Each dot represents the numbers of AASs (or harmful AASs) in each sample. The horizontal red bars indicate the median number of AASs (or harmful AASs) for each cancer type. n is the number of samples containing AASs (or harmful AASs) in each cancer type. The colored bars show the distribution of nucleotide substitutions in each sample. All the nucleotide substitutions are represented by pyrimidines at reference nucleotides. (PDF 692 kb) Additional file 3:
**This file contains supplementary Figure S2.** Distribution of predicted probabilities of harmfulness for cancer variants. a) Density plot of predicted probabilities of harmfulness for all AASs and most frequent AASs in COSMIC database. AASs present in more than 25 samples in COSMIC were referred as most frequent. The predicted probability ranges from 0–1 but the plots are extrapolated by default to 3 times the bandwidth from the extreme values. b) Density plot of predicted probabilities of harmfulness for somatic variation datasets obtained from ClinVar, DoCM and TP53 database. c) Venn diagram of overlap between somatic variations obtained from ClinVar, DoCM and TP53 database. d) Venn diagrams of overlap between frequent variations in COSMIC with somatic variations obtained from ClinVar, DoCM and TP53 database. (PDF 240 kb) Additional file 4:
**This file contains supplementary Figures S3 and S4.** Lego plots of base substitutions leading to AASs and harmful AASs in cancers. **Figure S3**: Lego plots of base substitutions leading to AASs in cancers. **Figure S4**: Lego plots of base substitutions leading to harmful AASs in cancers. The frequency is based on the variation and immediate 5’ and 3’ bases to the substituted nucleotide. The variations containing either 5’ or 3’ nucleotide in intronic region are excluded. The base substitutions are represented by six types of substitutions with pyrimidines at reference nucleotides. The color represents the frequency of each tri-nucleotide from the lowest (blue) to the highest (red). (PDF 728 kb) Additional file 5:
**This file contains supplementary Figures S5 and S6.** Lego plots of AASs and harmful AASs caused by single nucleotide substitutions in cancers. **Figure S5**: Lego plots of AASs caused by single nucleotide substitutions in cancers. **Figure S6**: Lego plots of AASs caused by single nucleotide substitutions in cancers. The color represents the frequency of each tri-nucleotide from the lowest (blue) to the highest (red). The most frequent AAS (s) are marked. (PDF 1140 kb) Additional file 6:
**This file contains supplementary Figure S7.** Distribution of AASs in protein domains. a, Frequency of AASs in protein domains. 20 domains containing the highest frequency of AASs in each cancer are plotted. b, Average frequency of AASs in protein domains in all cancers together. InterPro domains containing at least 2 AASs in at least one of the cancer types are included. (PDF 117 kb) Additional file 7:
**This file contains supplementary Figure S8.** Distribution of AASs in EGFR protein. a, Distribution of AASs along EGFR protein sequence in different cancer types. The X-axis represents the positions of the amino acids in the protein sequence. b, 3-dimensional protein structure of extracellular domain in EGFR (pdbid: 3QWQ). c, 3-dimensional protein structure of kinase domain in EGFR (pdbid: 3POZ). Positions of harmful AASs are highlighted in red, positions of benign AASs in blue and positions where both harmful and benign AASs are present in yellow. (PDF 215 kb) Additional file 8:
**This file contains supplementary Figure S9.** Numbers of genes containing SNVs leading to AASs in cancers. Numbers of genes corresponding to selected proteins, genes with at least one SNV leading to harmful AASs and genes with SNVs leading to AASs are shown, respectively, from left to right for each cancer type. The grey bar represents genes catalogued in CGC (genes in which SNVs leading to AASs have been implicated in cancer) and the white bar represents all other genes. (PDF 17 kb) Additional file 9:
**Contains supplementary Figures S10 to S37.** Pathway-based functional interaction network of proteins containing harmful AASs in 28 cancer types. Proteins involved in selected significantly enriched pathways are marked with colored backgrounds. The proteins were clustered into different modules by using ReactomeFI plugin in cytoscape. **Figure S10**: Acute lymphocytic leukemia (ALL). **Figure S11**: Acute myeloid leukemia (AML). **Figure S12**: Bladder cancer. **Figure S13**: Breast cancer. **Figure S14**: Cervix cancer. **Figure S15**: Chronic lymphocytic leukemia (CLL). **Figure S16**: Colorectum cancer. **Figure S17**: Esophageal cancer. **Figure S18**: Glioblastoma. **Figure S19**: Glioma low grade. **Figure S20**: Head and neck cancer (HNC). **Figure S21**: Kidney chromophobe cancer. **Figure S22**: Kidney clear cell cancer. **Figure S23**: Kidney papillary cancer. **Figure S24**: Liver cancer. **Figure S25**: Lung adeno cancer. **Figure S26**: Lung small cell cancer. **Figure S27**: Lung squamous cancer. **Figure S28**: Lymphoma B-cell. **Figure S29**: Melanoma cancer. **Figure S30**: Myeloma cancer. **Figure S31**: Neuroblastoma. **Figure S32**: Ovary cancer. **Figure S33**: Pancreas cancer. **Figure S34**: Prostate cancer. **Figure S35**: Stomach cancer. **Figure S36**: Thyroid cancer. **Figure S37**: Uterus cancer. (PDF 3125 kb) Additional file 10:
**Contains supplementary figures S38 and S39.** Pathway-based functional interaction network and significantly enriched pathways in ALL. **Figure S38**: Pathway-based functional interaction network in ALL. The proteins containing harmful AASs and proteins that are first neighbor of proteins containing more than one harmful AASs are included in the network. The network was clustered by using ReactomeFI plugin in cytoscape. The node colors represent clusters. **Figure S39**: Network of significantly enriched pathways in ALL. The nodes represent pathways and the edges represent overlapping proteins between the pathways and containing harmful AASs in ALL. The edge thickness represents the number of overlapping proteins. (PDF 813 kb)

## Abbreviations

AAS: Amino acid substitution
ALL: Acute lymphocytic leukemia
CGC: Cancer Gene Census
COSMIC: Catalogue of Somatic Mutation in Cancer
DoCM: Database of Curated Mutations
FDR: False discovery rate
GO: Gene Ontology
HNC: Head and neck cancer
SNV: Single nucleotide variation
