# Supplementary figures and images for: Harmful somatic amino acid substitutions affect key pathways in cancers
Source: BMC Med Genomics. 2015 Aug 19;8:53. doi: 10.1186/s12920-015-0125-x (PMC4539680; doi:10.1186/s12920-015-0125-x)

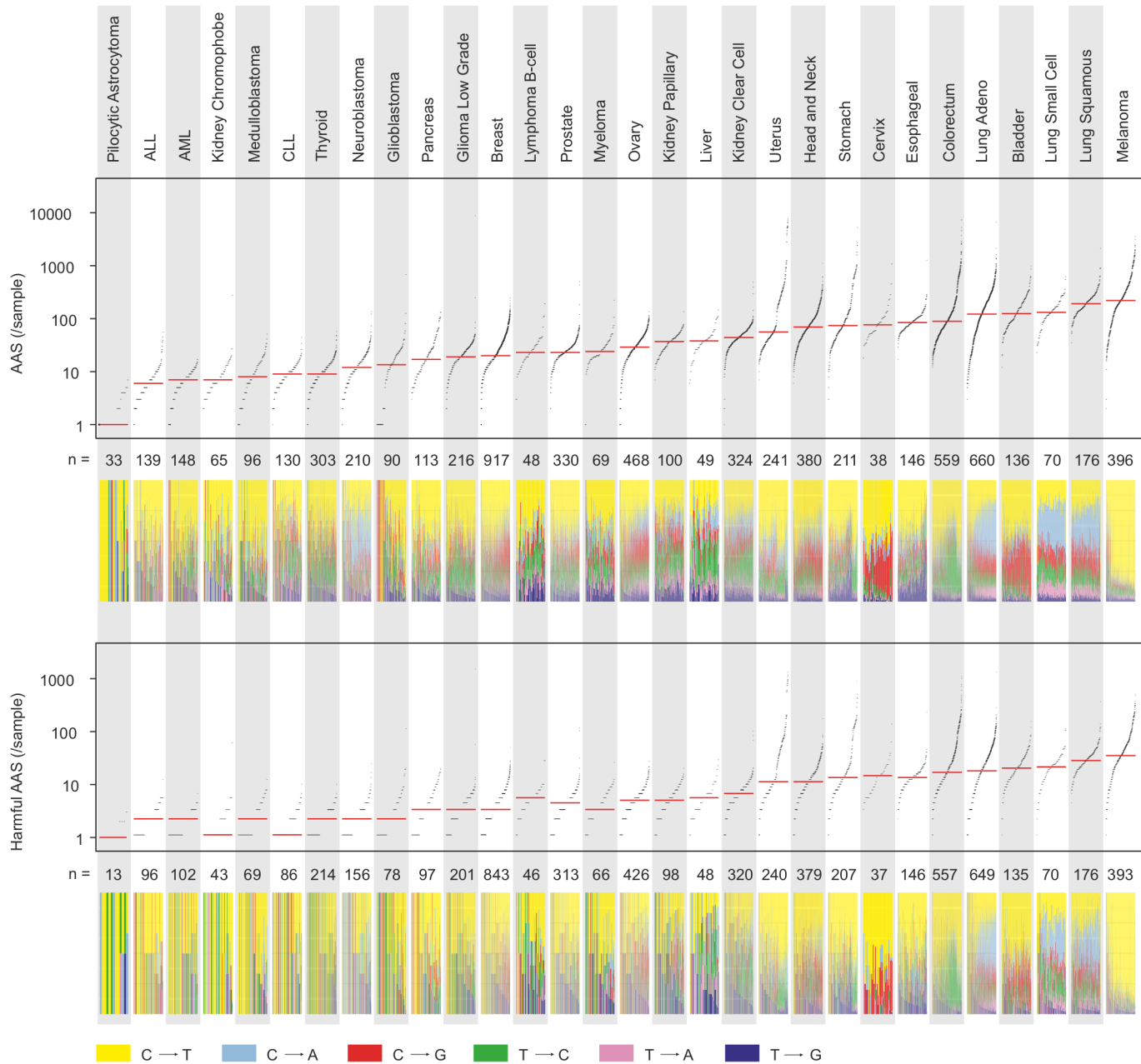

Supplement: Additional file 2: — This file contains supplementary Figure S1. Frequency of variations leading to AASs in each sample. Each dot represents the numbers of AASs (or harmful AASs) in each sample. The horizontal red bars indicate the median number of AASs (or harmful AASs) for each cancer type. n is the number of samples containing AASs (or harmful AASs) in each cancer type. The colored bars show the distribution of nucleotide substitutions in each sample. All the nucleotide substitutions are represented by pyrimidines at reference nucleotides. (PDF 692 kb) [file 12920_2015_125_MOESM2_ESM.pdf]

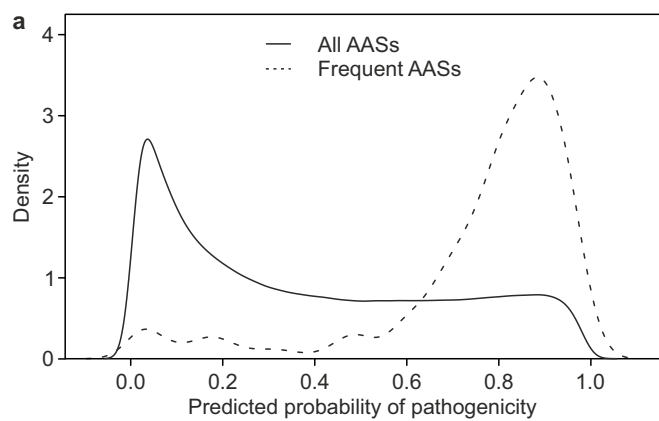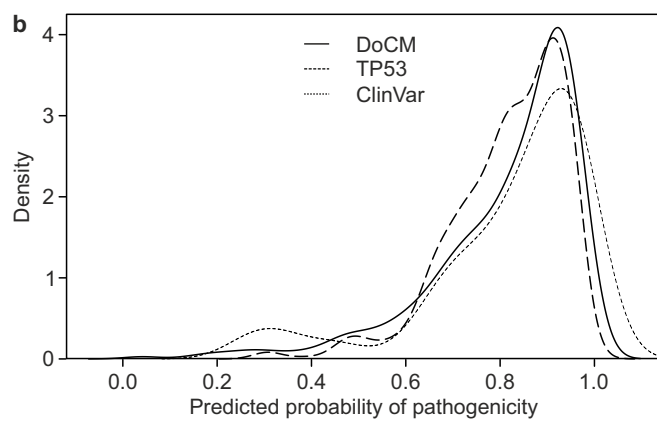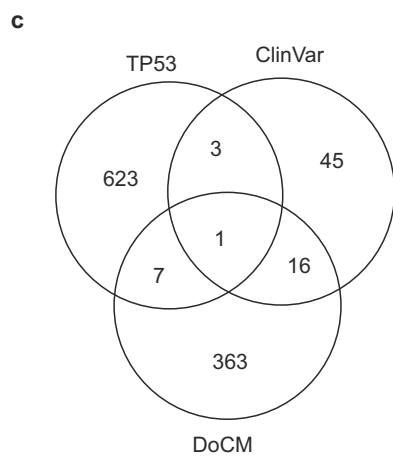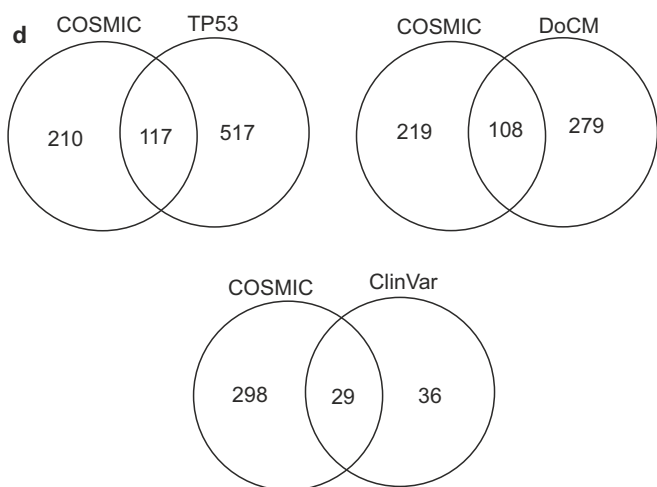

Supplement: Additional file 3: — This file contains supplementary Figure S2. Distribution of predicted probabilities of harmfulness for cancer variants. a) Density plot of predicted probabilities of harmfulness for all AASs and most frequent AASs in COSMIC database. AASs present in more than 25 samples in COSMIC were referred as most frequent. The predicted probability ranges from 0–1 but the plots are extrapolated by default to 3 times the bandwidth from the extreme values. b) Density plot of predicted probabilities of harmfulness for somatic variation datasets obtained from ClinVar, DoCM and TP53 database. c) Venn diagram of overlap between somatic variations obtained from ClinVar, DoCM and TP53 database. d) Venn diagrams of overlap between frequent variations in COSMIC with somatic variations obtained from ClinVar, DoCM and TP53 database. (PDF 240 kb) [file 12920_2015_125_MOESM3_ESM.pdf]

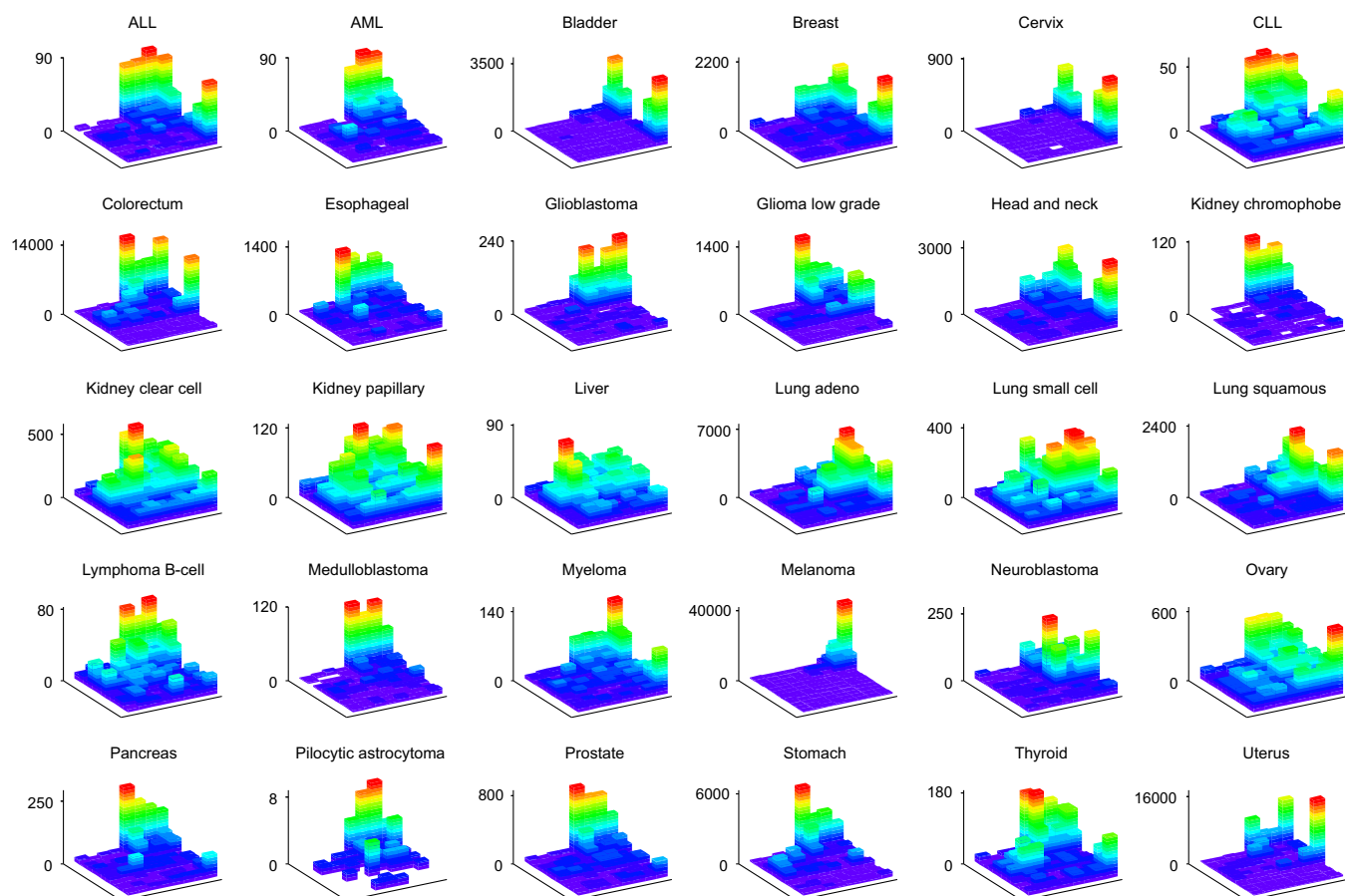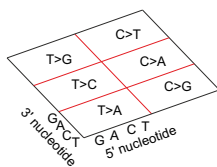

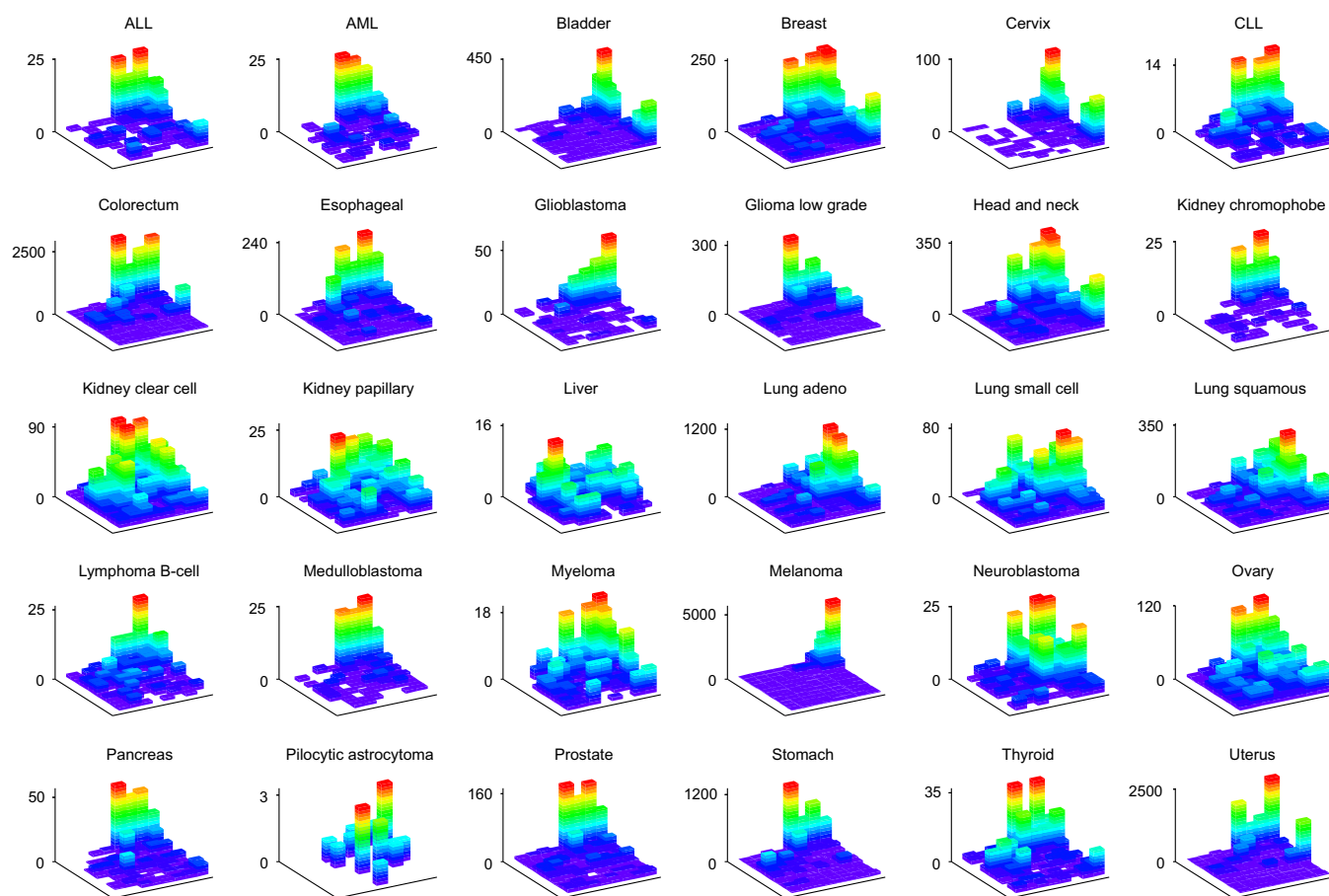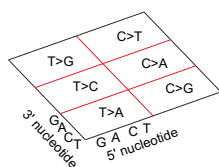

Supplement: Additional file 4: — This file contains supplementary Figures S3 and S4. Lego plots of base substitutions leading to AASs and harmful AASs in cancers. Figure S3: Lego plots of base substitutions leading to AASs in cancers. Figure S4: Lego plots of base substitutions leading to harmful AASs in cancers. The frequency is based on the variation and immediate 5’ and 3’ bases to the substituted nucleotide. The variations containing either 5’ or 3’ nucleotide in intronic region are excluded. The base substitutions are represented by six types of substitutions with pyrimidines at reference nucleotides. The color represents the frequency of each tri-nucleotide from the lowest (blue) to the highest (red). (PDF 728 kb) [file 12920_2015_125_MOESM4_ESM.pdf]

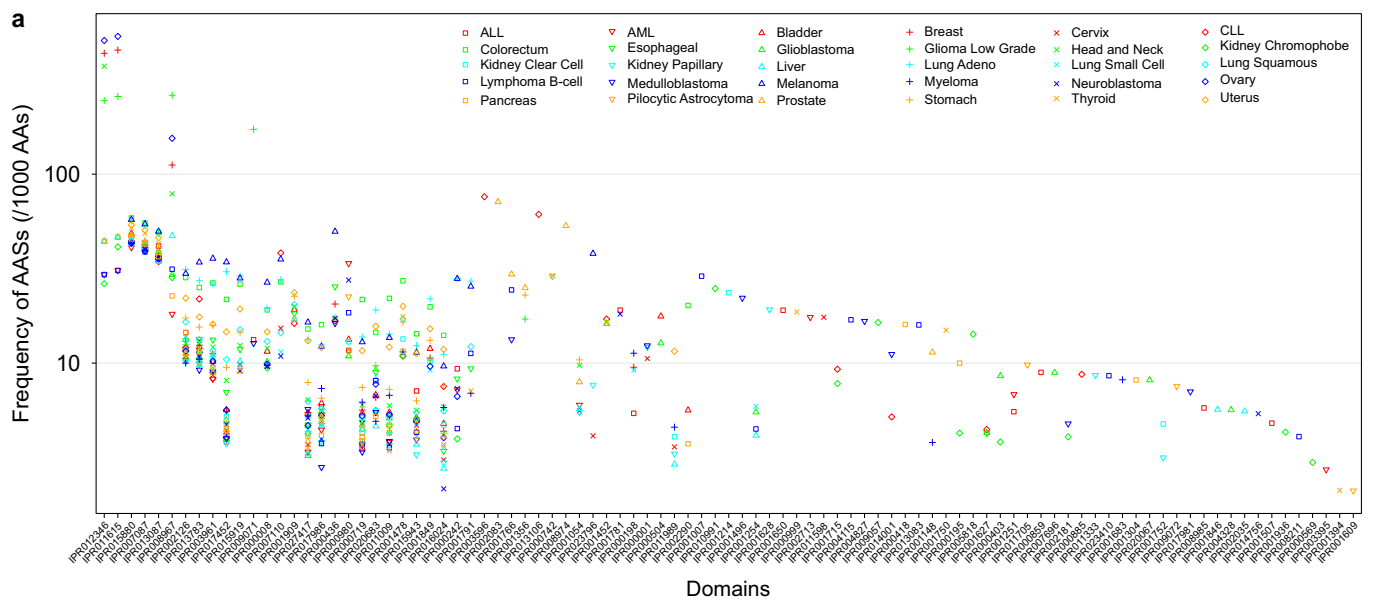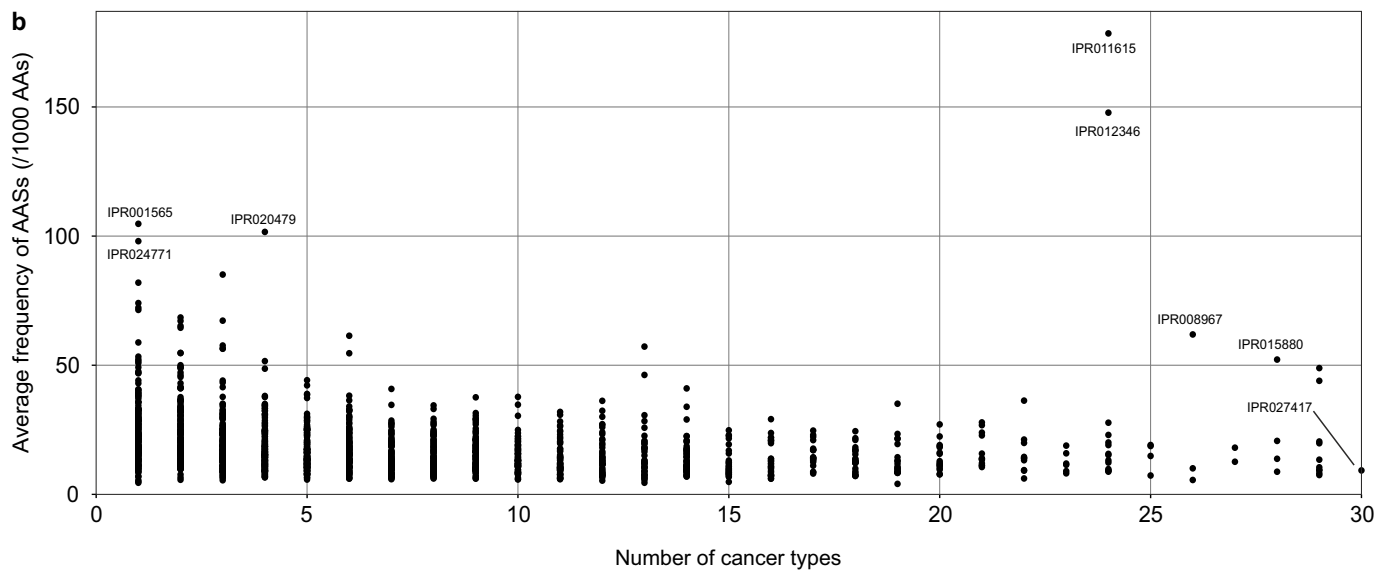

Supplement: Additional file 6: — This file contains supplementary Figure S7. Distribution of AASs in protein domains. a, Frequency of AASs in protein domains. 20 domains containing the highest frequency of AASs in each cancer are plotted. b, Average frequency of AASs in protein domains in all cancers together. InterPro domains containing at least 2 AASs in at least one of the cancer types are included. (PDF 117 kb) [file 12920_2015_125_MOESM6_ESM.pdf]

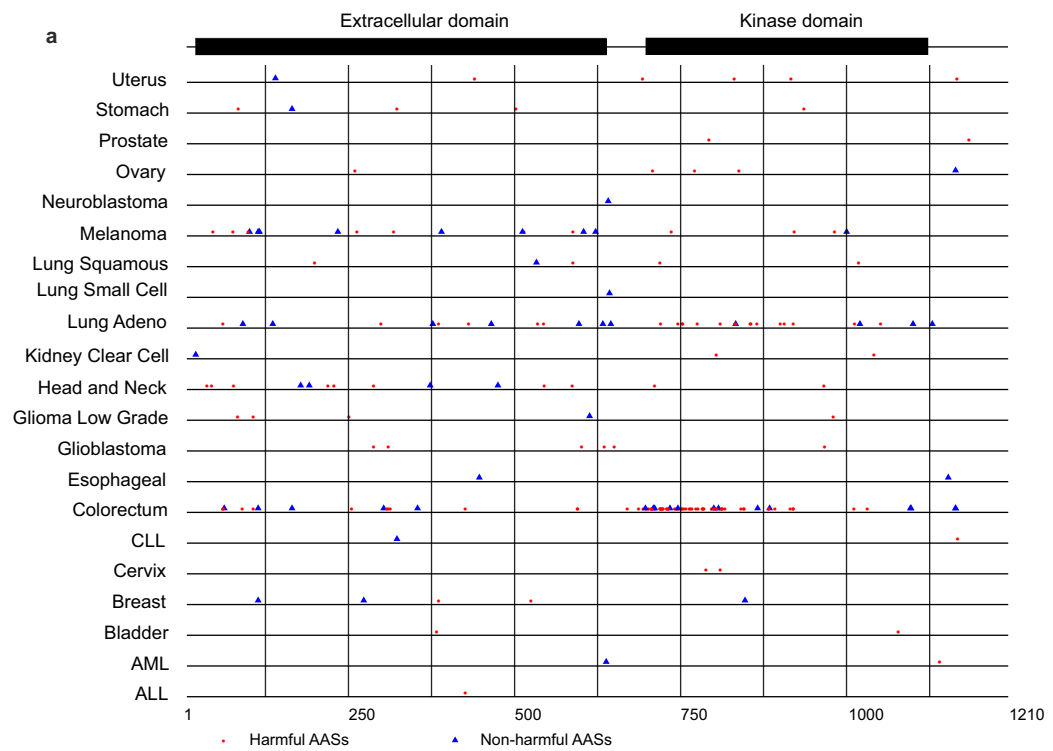

**b**

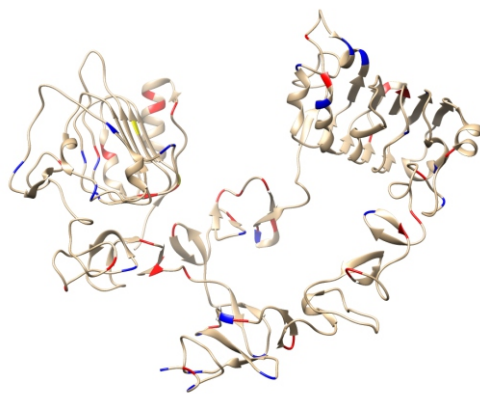

**c**

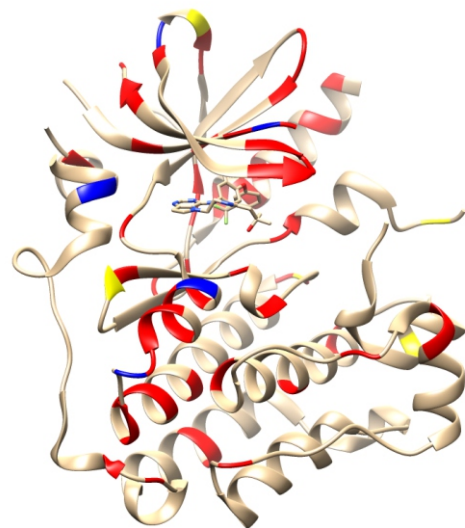

Supplement: Additional file 7: — This file contains supplementary Figure S8. Distribution of AASs in EGFR protein. a, Distribution of AASs along EGFR protein sequence in different cancer types. The X-axis represents the positions of the amino acids in the protein sequence. b, 3-dimensional protein structure of extracellular domain in EGFR (pdbid: 3QWQ). c, 3-dimensional protein structure of kinase domain in EGFR (pdbid: 3POZ). Positions of harmful AASs are highlighted in red, positions of benign AASs in blue and positions where both harmful and benign AASs are present in yellow. (PDF 215 kb) [file 12920_2015_125_MOESM7_ESM.pdf]

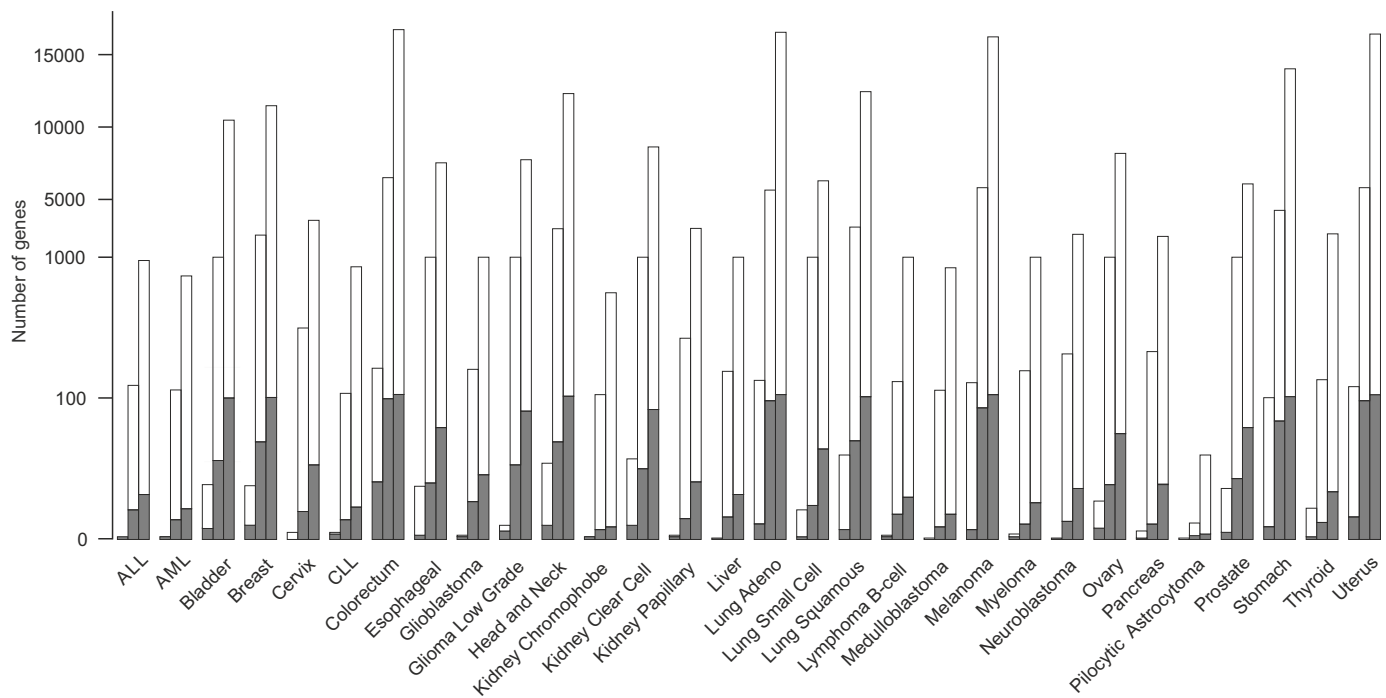

Supplement: Additional file 8: — This file contains supplementary Figure S9. Numbers of genes containing SNVs leading to AASs in cancers. Numbers of genes corresponding to selected proteins, genes with at least one SNV leading to harmful AASs and genes with SNVs leading to AASs are shown, respectively, from left to right for each cancer type. The grey bar represents genes catalogued in CGC (genes in which SNVs leading to AASs have been implicated in cancer) and the white bar represents all other genes. (PDF 17 kb) [file 12920_2015_125_MOESM8_ESM.pdf]

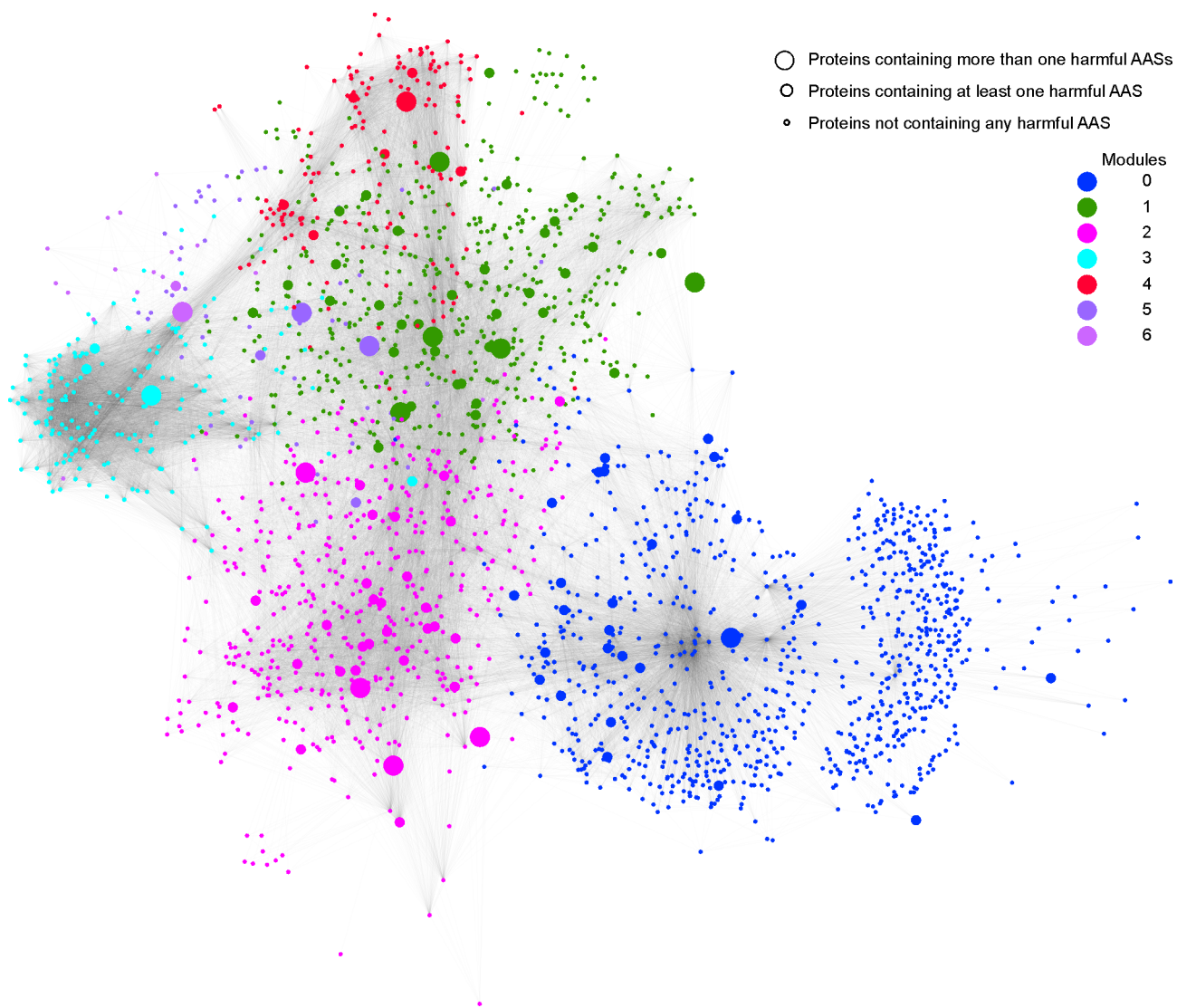

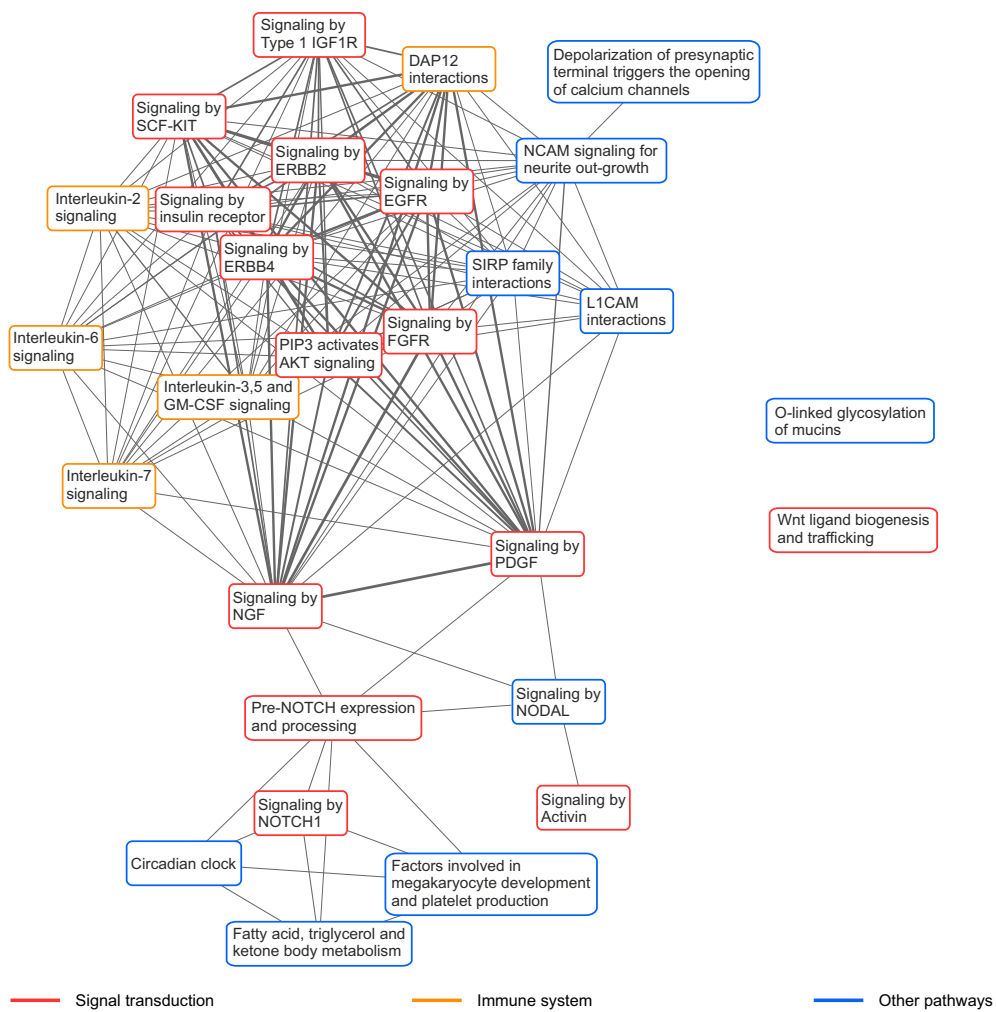

Supplement: Additional file 10: — Contains supplementary figures S38 and S39. Pathway-based functional interaction network and significantly enriched pathways in ALL. Figure S38: Pathway-based functional interaction network in ALL. The proteins containing harmful AASs and proteins that are first neighbor of proteins containing more than one harmful AASs are included in the network. The network was clustered by using ReactomeFI plugin in cytoscape. The node colors represent clusters. Figure S39: Network of significantly enriched pathways in ALL. The nodes represent pathways and the edges represent overlapping proteins between the pathways and containing harmful AASs in ALL. The edge thickness represents the number of overlapping proteins. (PDF 813 kb) [file 12920_2015_125_MOESM10_ESM.pdf]
