# Supplementary material for: Harmful somatic amino acid substitutions affect key pathways in cancers
Source: BMC Med Genomics. 2015 Aug 19;8:53. doi: 10.1186/s12920-015-0125-x (PMC4539680; doi:10.1186/s12920-015-0125-x)
Supplement: Additional file 5: — This file contains supplementary Figures S5 and S6. Lego plots of AASs and harmful AASs caused by single nucleotide substitutions in cancers. Figure S5: Lego plots of AASs caused by single nucleotide substitutions in cancers. Figure S6: Lego plots of AASs caused by single nucleotide substitutions in cancers. The color represents the frequency of each tri-nucleotide from the lowest (blue) to the highest (red). The most frequent AAS (s) are marked. (PDF 1140 kb) [file 12920_2015_125_MOESM5_ESM.pdf]

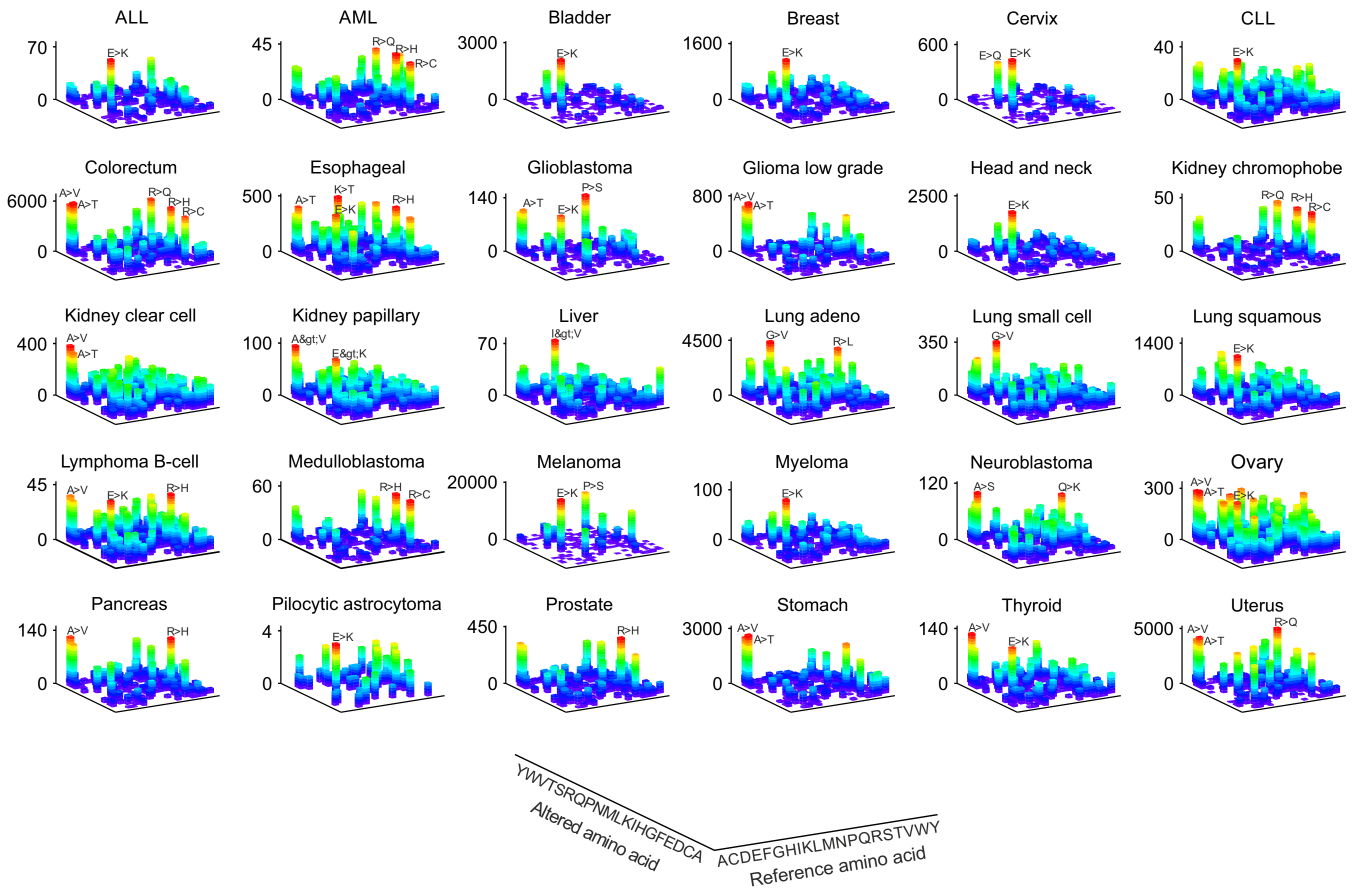

ALL
70
0
E>K
AML
45
0
R>Q
R>H
R>C
Bladder
3000
0
E>K
Breast
1600
0
E>K
Cervix
600
0
E>Q
E>K
CLL
40
0
E>K
Colorectum
6000
0
A>V
A>T
R>Q
R>H
R>C
Esophageal
500
0
A>T
K>T
E>K
R>H
Glioblastoma
140
0
A>T
E>K
P>S
Glioma low grade
800
0
A>V
A>T
Head and neck
2500
0
E>K
Kidney chromophobe
50
0
R>Q
R>H
R>C
Kidney clear cell
400
0
A>V
A>T
Kidney papillary
100
0
A>V
E>K
Liver
70
0
I>V
Lung adeno
4500
0
G>V
R>L
Lung small cell
350
0
G>V
Lung squamous
1400
0
E>K
Lymphoma B-cell
45
0
A>V
E>K
R>H
Medulloblastoma
60
0
R>H
R>C
Melanoma
20000
0
E>K
P>S
Myeloma
100
0
E>K
Neuroblastoma
120
0
A>S
Q>K
Ovary
300
0
A>V
A>T
E>K
Pancreas
140
0
A>V
R>H
Pilocytic astrocytoma
4
0
E>K
Prostate
450
0
R>H
Stomach
3000
0
A>V
A>T
Thyroid
140
0
A>V
E>K
Uterus
5000
0
A>V
A>T
R>Q
YWVTSRQPNMLKIHGFEDCA
Altered amino acid
ACDEFGHIKLMNPQRSTVWY
Reference amino acid

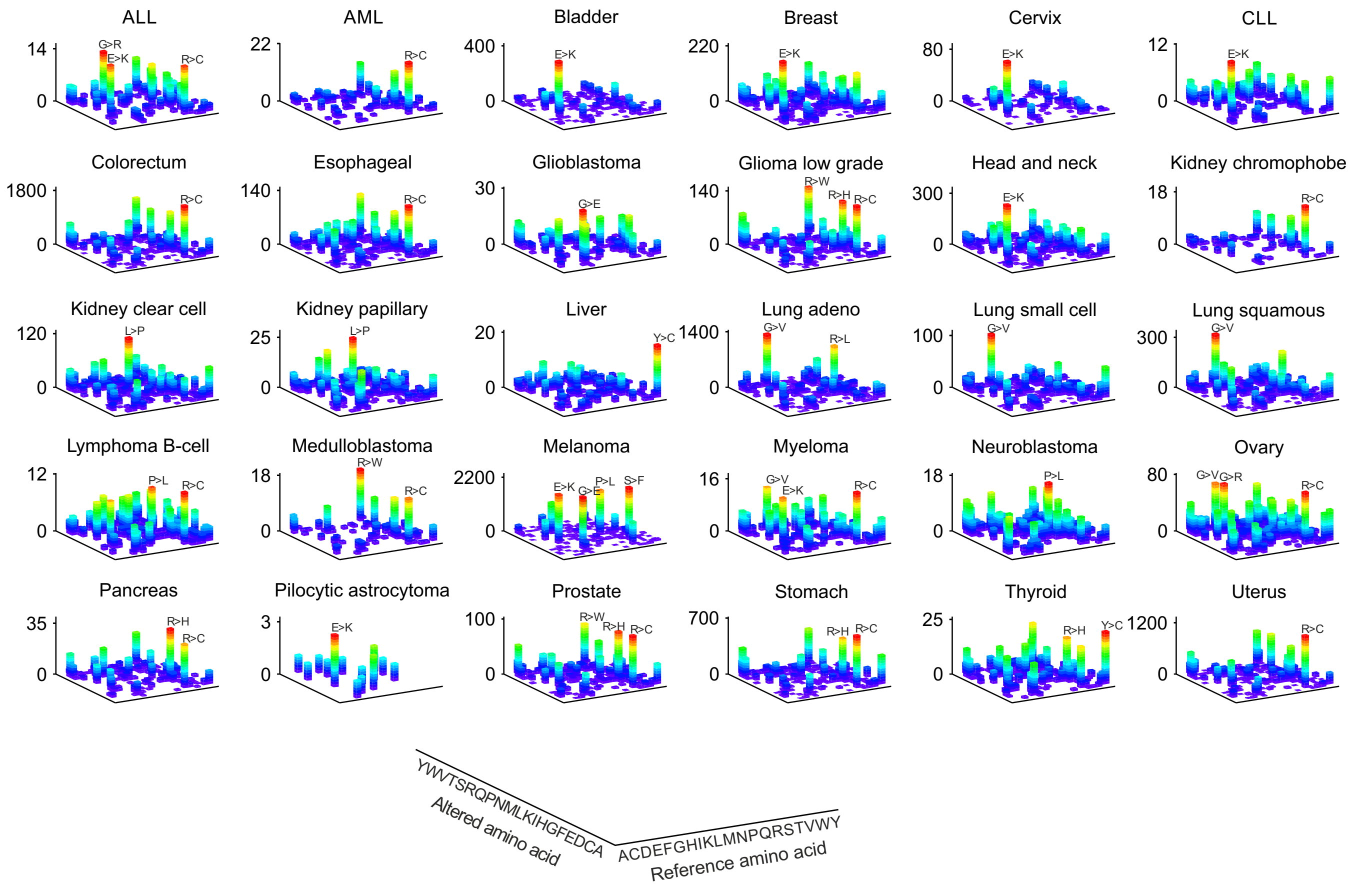
ALL
AML
Bladder
Breast
Cervix
CLL
Colorectum
Esophageal
Glioblastoma
Glioma low grade
Head and neck
Kidney chromophobe
Kidney clear cell
Kidney papillary
Liver
Lung adeno
Lung small cell
Lung squamous
Lymphoma B-cell
Medulloblastoma
Melanoma
Myeloma
Neuroblastoma
Ovary
Pancreas
Pilocytic astrocytoma
Prostate
Stomach
Thyroid
Uterus
YWVTSRQPNMLKIHGFEDCA
Altered amino acid
ACDEFGHIKLMNPQRSTVWY
Reference amino acid
