# Supplementary material for: Harmful somatic amino acid substitutions affect key pathways in cancers
Source: BMC Med Genomics. 2015 Aug 19;8:53. doi: 10.1186/s12920-015-0125-x (PMC4539680; doi:10.1186/s12920-015-0125-x)
Supplement: Additional file 9: — Contains supplementary Figures S10 to S37. Pathway-based functional interaction network of proteins containing harmful AASs in 28 cancer types. Proteins involved in selected significantly enriched pathways are marked with colored backgrounds. The proteins were clustered into different modules by using ReactomeFI plugin in cytoscape. Figure S10: Acute lymphocytic leukemia (ALL). Figure S11: Acute myeloid leukemia (AML). Figure S12: Bladder cancer. Figure S13: Breast cancer. Figure S14: Cervix cancer. Figure S15: Chronic lymphocytic leukemia (CLL). Figure S16: Colorectum cancer. Figure S17: Esophageal cancer. Figure S18: Glioblastoma. Figure S19: Glioma low grade. Figure S20: Head and neck cancer (HNC). Figure S21: Kidney chromophobe cancer. Figure S22: Kidney clear cell cancer. Figure S23: Kidney papillary cancer. Figure S24: Liver cancer. Figure S25: Lung adeno cancer. Figure S26: Lung small cell cancer. Figure S27: Lung squamous cancer. Figure S28: Lymphoma B-cell. Figure S29: Melanoma cancer. Figure S30: Myeloma cancer. Figure S31: Neuroblastoma. Figure S32: Ovary cancer. Figure S33: Pancreas cancer. Figure S34: Prostate cancer. Figure S35: Stomach cancer. Figure S36: Thyroid cancer. Figure S37: Uterus cancer. (PDF 3125 kb) [file 12920_2015_125_MOESM9_ESM.pdf]

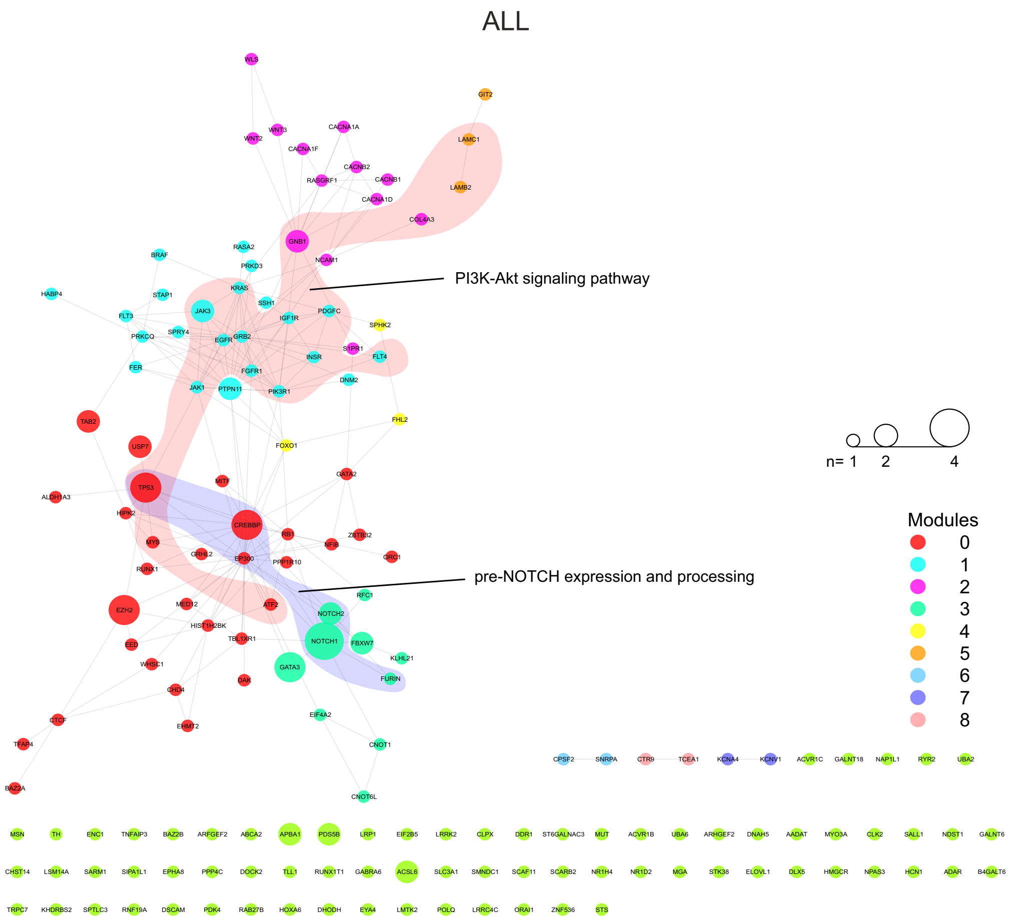
ALL
PI3K-Akt signaling pathway
pre-NOTCH expression and processing
n= 1
2
4
Modules
0
1
2
3
4
5
6
7
8

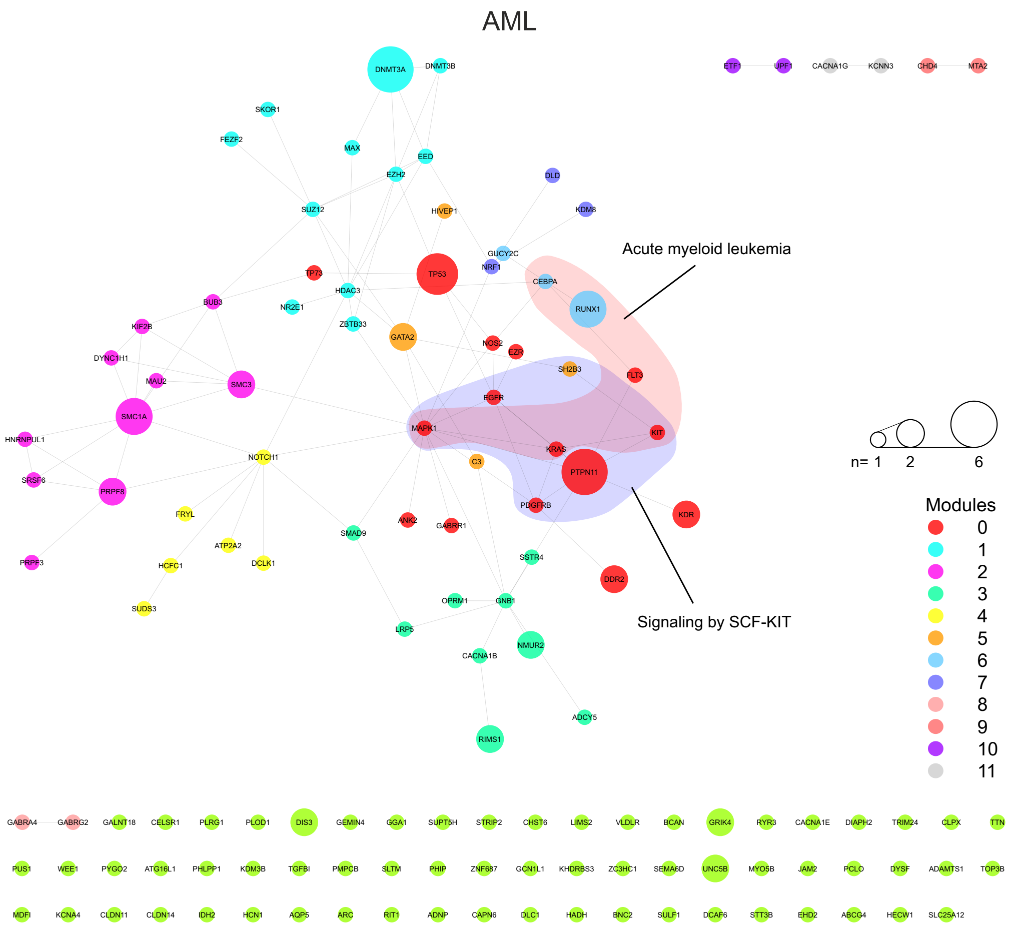
AML
Acute myeloid leukemia
Signaling by SCF-KIT
n= 1 2 6
Modules
0
1
2
3
4
5
6
7
8
9
10
11
DNMT3A
DNMT3B
SKOR1
FEZF2
MAX
EED
EZH2
DLD
SUZ12
HIVEP1
KDM8
GUCY2C
NRF1
TP73
TP53
HDAC3
CEBPA
RUNX1
BUB3
NR2E1
ZBTB33
KIF2B
GATA2
NOS2
EZR
DYNC1H1
SH2B3
FLT3
MAU2
SMC3
EGFR
SMC1A
MAPK1
KIT
HNRNPUL1
NOTCH1
KRAS
C3
PTPN11
SRSF6
PRPF8
PDGFRB
KDR
FRYL
ANK2
GABRR1
SMAD9
ATP2A2
SSTR4
PRPF3
HCFC1
DCLK1
DDR2
SUDS3
OPRM1
GNB1
LRP5
NMUR2
CACNA1B
ADCY5
RIMS1
ETF1
UPF1
CACNA1G
KCNN3
CHD4
MTA2
GABRA4
GABRG2
GALNT18
CELSR1
PLRG1
PLOD1
DIS3
GEMIN4
GGA1
SUPT5H
STRIP2
CHST6
LIMS2
VLDLR
BCAN
GRIK4
RYR3
CACNA1E
DIAPH2
TRIM24
CLPX
TTN
PUS1
WEE1
PYGO2
ATG16L1
PHLPP1
KDM3B
TGFBI
PMPCB
SLTM
PHIP
ZNF687
GCN1L1
KHDRBS3
ZC3HC1
SEMA6D
UNC5B
MYO5B
JAM2
PCLO
DYSF
ADAMTS1
TOP3B
MDFI
KCNA4
CLDN11
CLDN14
IDH2
HCN1
AQP5
ARC
RIT1
ADNP
CAPN6
DLC1
HADH
BNC2
SULF1
DCAF6
STT3B
EHD2
ABCG4
HECW1
SLC25A12

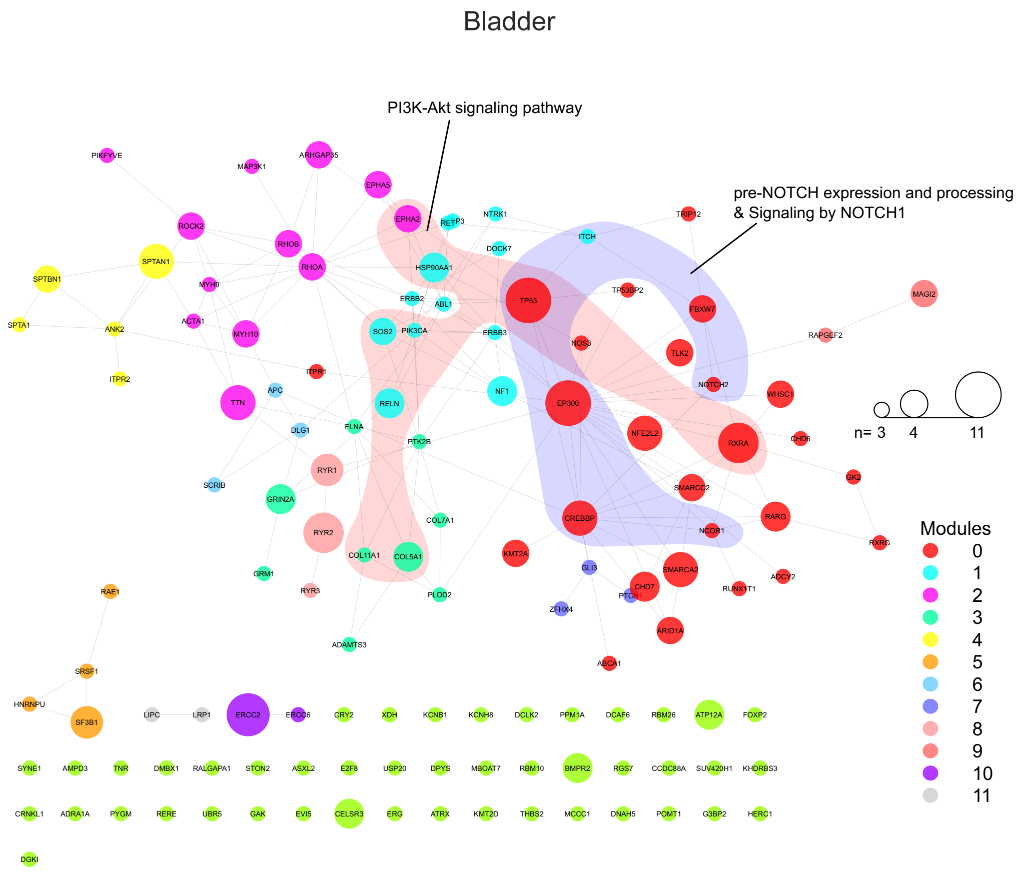

Bladder
PI3K-Akt signaling pathway
pre-NOTCH expression and processing
& Signaling by NOTCH1
PIKFYVE
MAP3K1
ARHGAP35
EPHA5
EPHA2
RETP3
NTRK1
TRIP12
ROCK2
RHOB
ITCH
DOCK7
SPTAN1
RHOA
HSP90AA1
SPTBN1
MYH9
TP53BP2
MAGI2
ERBB2
ABL1
TP53
FBXW7
SPTA1
ANK2
ACTA1
MYH10
SOS2
PIK3CA
ERBB3
RAPGEF2
NOS3
TLK2
ITPR2
ITPR1
NOTCH2
APC
NF1
WHSC1
TTN
RELN
EP300
DLG1
FLNA
NFE2L2
RXRA
CHD6
PTK2B
RYR1
GK2
SCRIB
GRIN2A
SMARCC2
COL7A1
CREBBP
RARG
RYR2
NCOR1
RXRG
COL11A1
COL5A1
KMT2A
GLI3
SMARCA2
ADCY2
GRM1
CHD7
RUNX1T1
RAE1
RYR3
PLOD2
PTCH1
ZFHX4
ARID1A
ADAMTS3
ABCA1
SRSF1
HNRNPU
SF3B1
LIPC
LRP1
ERCC2
ERCC6
CRY2
XDH
KCNB1
KCNH8
DCLK2
PPM1A
DCAF6
RBM26
ATP12A
FOXP2
SYNE1
AMPD3
TNR
DMBX1
RALGAPA1
STON2
ASXL2
E2F8
USP20
DPYS
MBOAT7
RBM10
BMPR2
RGS7
CCDC88A
SUV420H1
KHDRBS3
CRNKL1
ADRA1A
PYGM
RERE
UBR5
GAK
EVI5
CELSR3
ERG
ATRX
KMT2D
THBS2
MCCC1
DNAH5
POMT1
G3BP2
HERC1
DGKI
n= 3
4
11
Modules
0
1
2
3
4
5
6
7
8
9
10
11

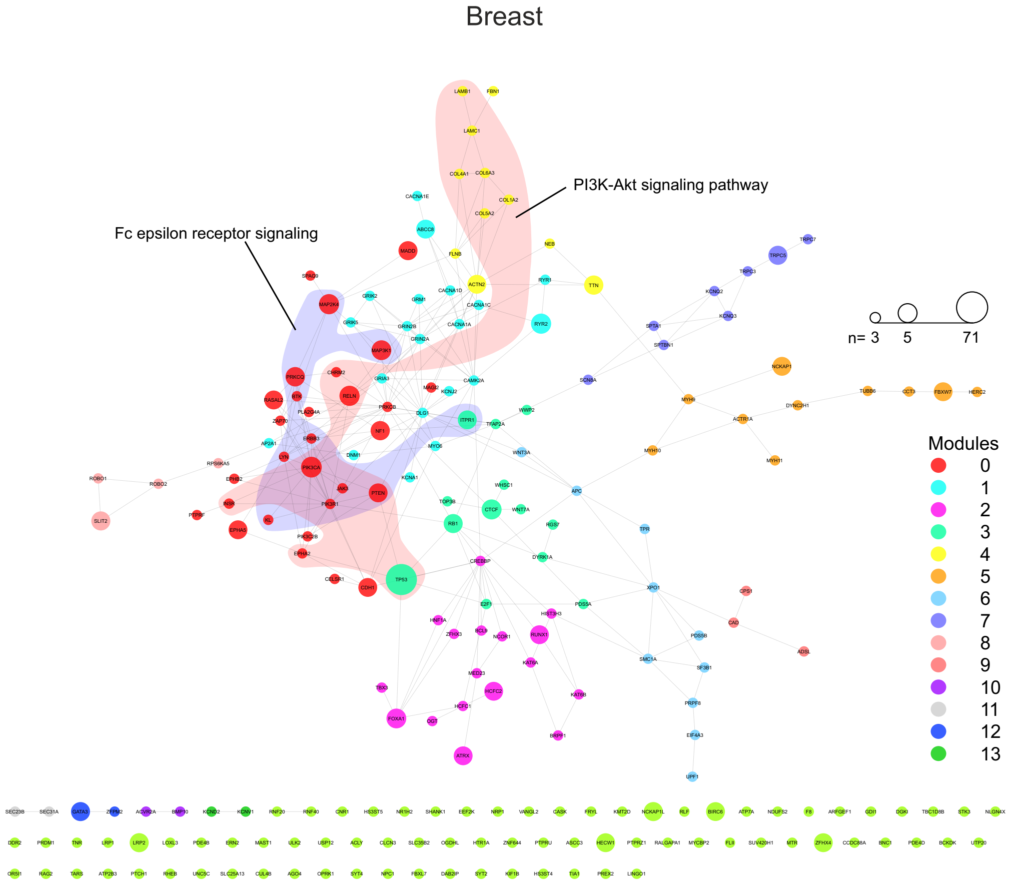

Breast
PI3K-Akt signaling pathway
Fc epsilon receptor signaling
n= 3
5
71
Modules
0
1
2
3
4
5
6
7
8
9
10
11
12
13

Cervix

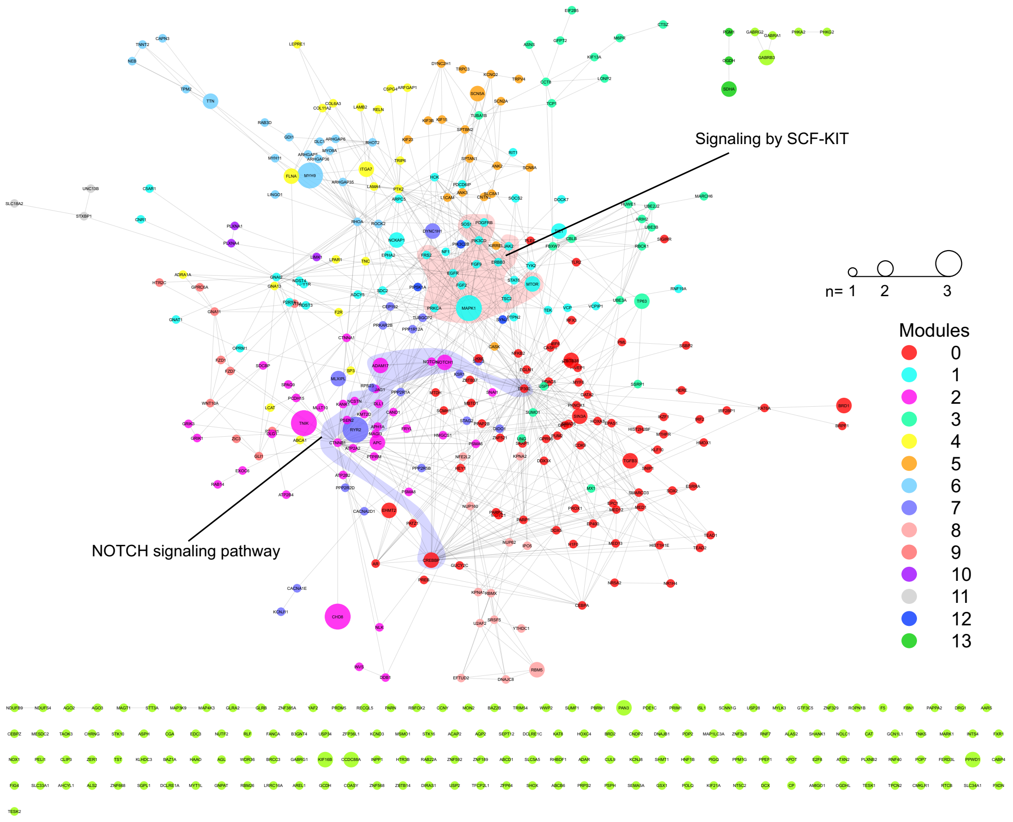

Signaling by SCF-KIT
NOTCH signaling pathway
n= 1
2
3
Modules
0
1
2
3
4
5
6
7
8
9
10
11
12
13

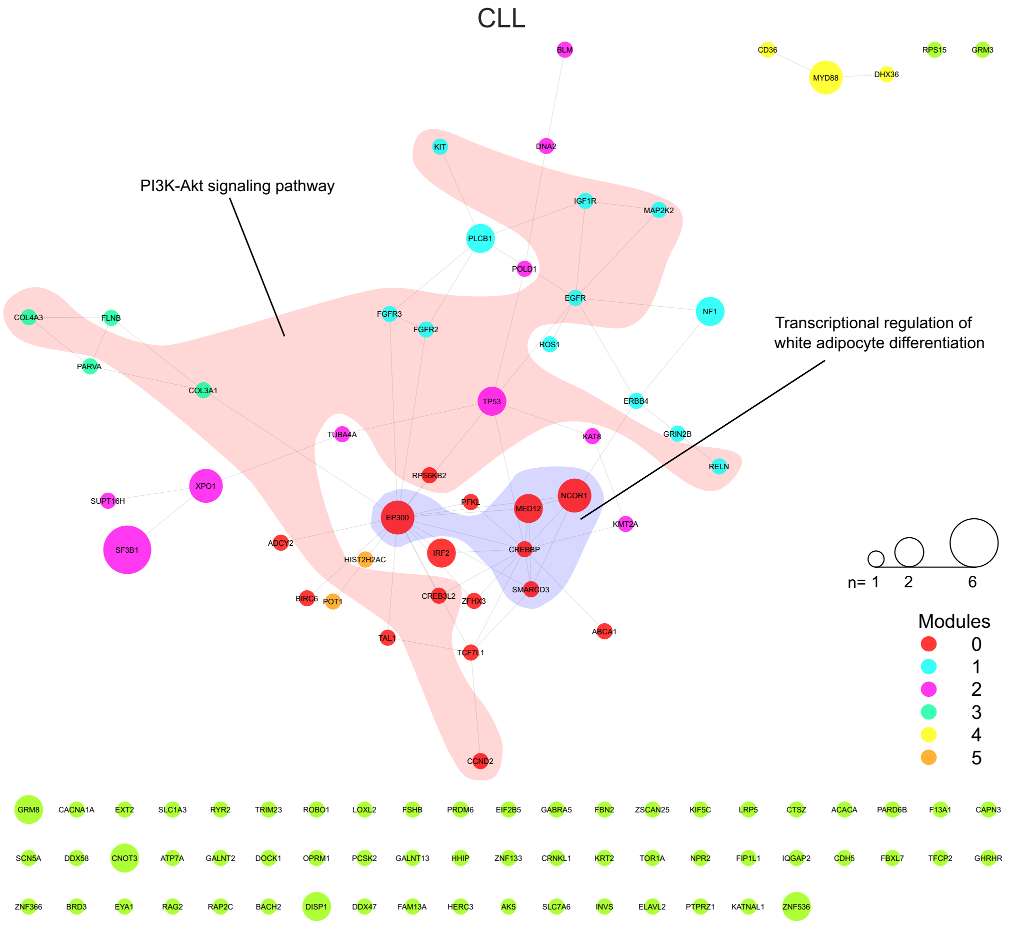
CLL
BLM
CD36
MYD88
DHX36
RPS15
GRM3
KIT
DNA2
PI3K-Akt signaling pathway
IGF1R
MAP2K2
PLCB1
POLD1
EGFR
NF1
COL4A3
FLNB
FGFR3
FGFR2
ROS1
Transcriptional regulation of
white adipocyte differentiation
PARVA
COL3A1
TP53
ERBB4
TUBA4A
KAT8
GRIN2B
RELN
RPS6KB2
SUPT16H
XPO1
PFKL
MED12
NCOR1
EP300
KMT2A
SF3B1
ADCY2
IRF2
CREBBP
HIST2H2AC
SMARCD3
CREB3L2
ZFHX3
BIRC6
POT1
ABCA1
TAL1
TCF7L1
CCND2
n= 1
2
6
Modules
0
1
2
3
4
5
GRM8
CACNA1A
EXT2
SLC1A3
RYR2
TRIM23
ROBO1
LOXL2
FSHB
PRDM6
EIF2B5
GABRA5
FBN2
ZSCAN25
KIF5C
LRP5
CTSZ
ACACA
PARD6B
F13A1
CAPN3
SCN5A
DDX58
CNOT3
ATP7A
GALNT2
DOCK1
OPRM1
PCSK2
GALNT13
HHIP
ZNF133
CRNKL1
KRT2
TOR1A
NPR2
FIP1L1
IQGAP2
CDH5
FBXL7
TFCP2
GHRHR
ZNF366
BRD3
EYA1
RAG2
RAP2C
BACH2
DISP1
DDX47
FAM13A
HERC3
AK5
SLC7A6
INVS
ELAVL2
PTPRZ1
KATNAL1
ZNF536

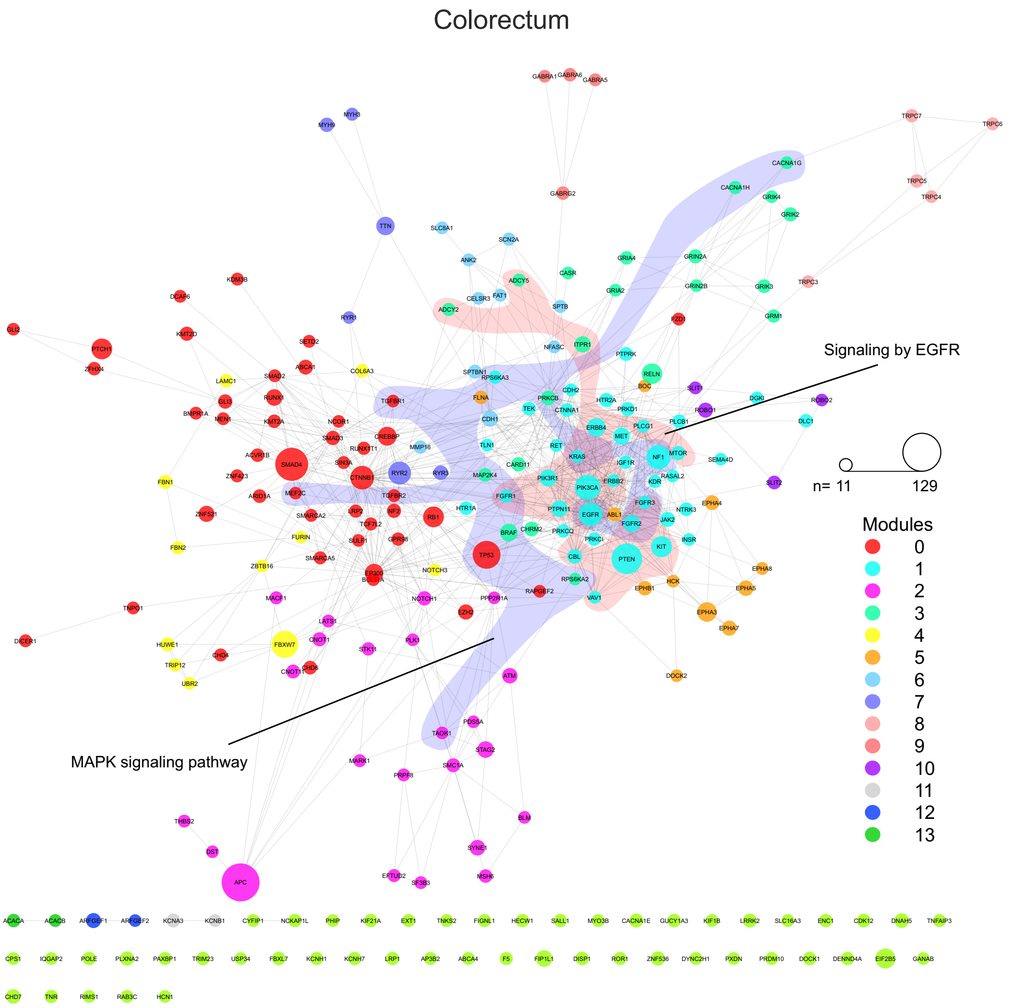
Colorectum
Signaling by EGFR
MAPK signaling pathway
n= 11
129
Modules
0
1
2
3
4
5
6
7
8
9
10
11
12
13

Esophageal

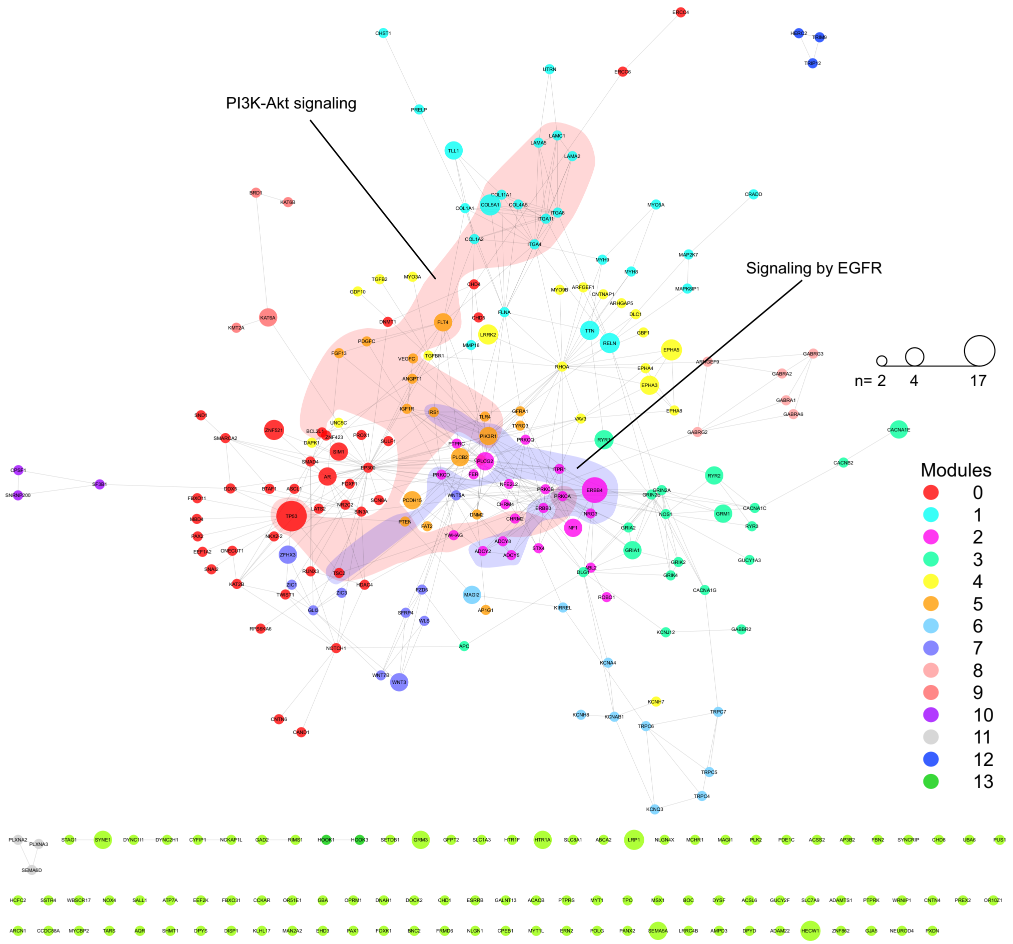
PI3K-Akt signaling
Signaling by EGFR
n= 2
4
17
Modules
0
1
2
3
4
5
6
7
8
9
10
11
12
13

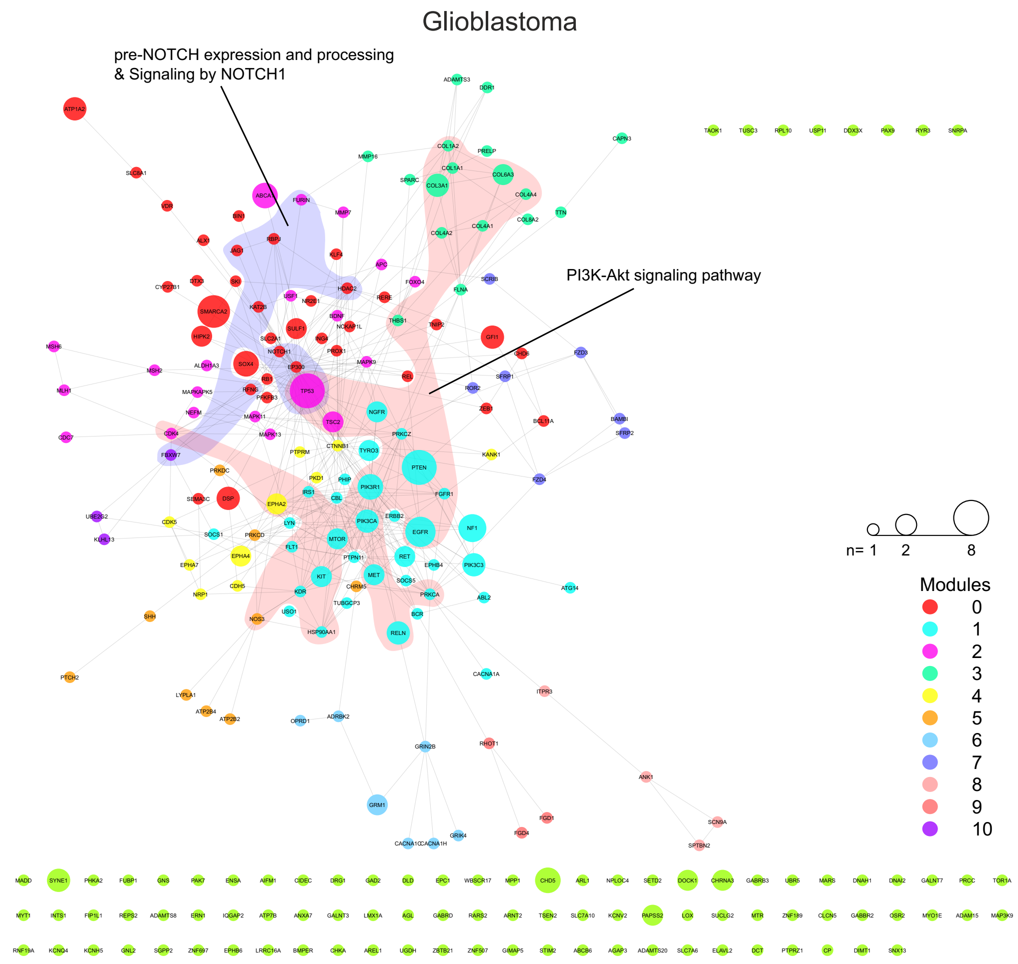

Glioblastoma
pre-NOTCH expression and processing
& Signaling by NOTCH1
PI3K-Akt signaling pathway
n= 1
2
8
Modules
0
1
2
3
4
5
6
7
8
9
10

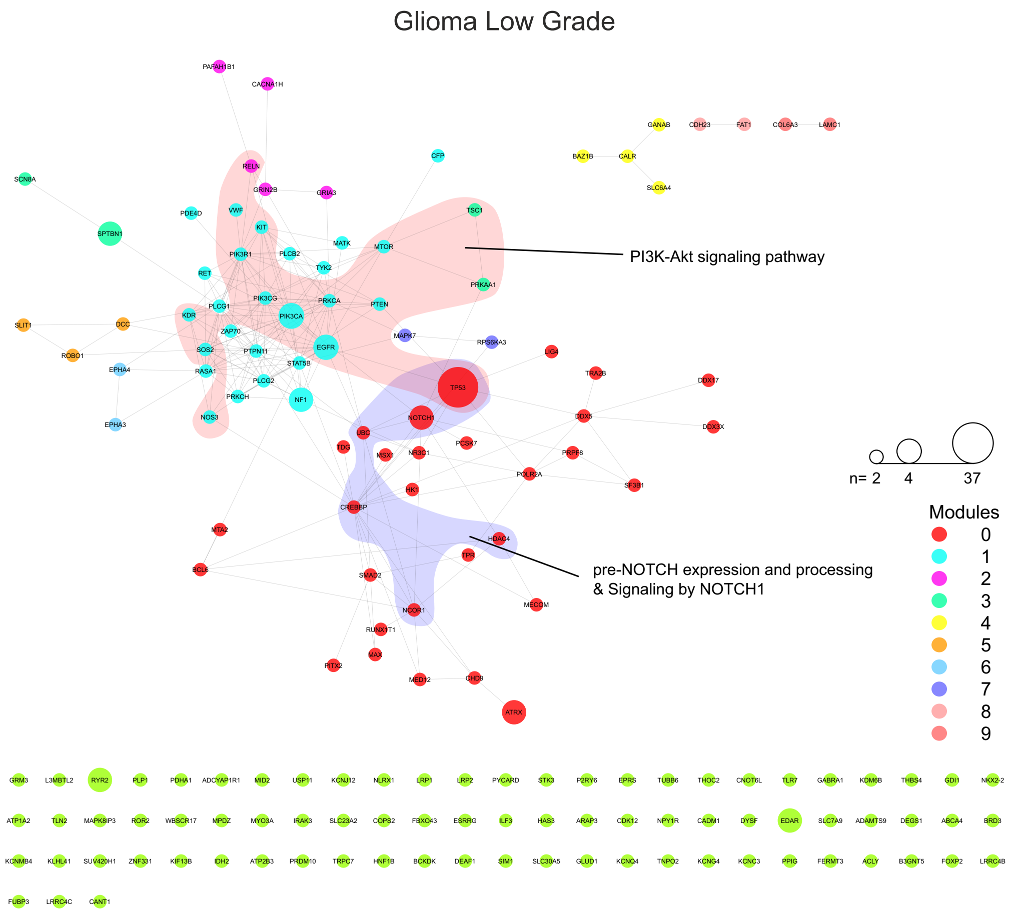

Glioma Low Grade
PAFAH1B1
CACNA1H
RELN
GRIN2B
GRIA3
CFP
SCN8A
PDE4D
VWF
KIT
SPTBN1
MATK
MTOR
TSC1
PIK3R1
PLCB2
TYK2
RET
PRKAA1
PIK3CG
PRKCA
PTEN
PLCG1
PIK3CA
KDR
SLIT1
DCC
ZAP70
MAPK7
RPS6KA3
SOS2
PTPN11
EGFR
LIG4
ROBO1
STAT5B
EPHA4
RASA1
PLCG2
TP63
TRA2B
DDX17
PRKCH
NF1
NOTCH1
DDX5
NOS3
EPHA3
DDX3X
UBC
PCSK7
TDG
NSX1
NR3C1
PRPF8
POLR2A
HK1
SF3B1
CREBBP
MTA2
HDAC4
TPR
BCL6
SMAD2
MECOM
NCOR1
RUNX1T1
MAX
PITX2
MED12
CHD9
ATRX
GANAB
CDH23
FAT1
COL6A3
LAMC1
BAZ1B
CALR
SLC6A4
PI3K-Akt signaling pathway
pre-NOTCH expression and processing & Signaling by NOTCH1
n= 2
4
37
Modules
0
1
2
3
4
5
6
7
8
9
GRM3
L3MBTL2
RYR2
PLP1
PDHA1
ADCYAP1R1
MID2
USP11
KCNJ12
NLRX1
LRP1
LRP2
PYCARD
STK3
P2RY6
EPRS
TUBB6
THOC2
CNOT6L
TLR7
GABRA1
KDM6B
THBS4
GDI1
NKX2-2
ATP1A2
TLN2
MAPK8IP3
ROR2
WBSCR17
MPDZ
MYO3A
IRAK3
SLC23A2
COPS2
FBXO43
ESRRG
ILF3
HAS3
ARAP3
CDK12
NPY1R
CADM1
DYSF
EDAR
SLC7A9
ADAMTS9
DEGS1
ABCA4
BRD3
KCNMB4
KLHL41
SUV420H1
ZNF331
KIF13B
IDH2
ATP2B3
PRDM10
TRPC7
HNF1B
BCKDK
DEAF1
SIM1
SLC30A5
GLUD1
KCNQ4
TNPO2
KCNG4
KCNC3
PPIG
FERMT3
ACLY
B3GNT5
FOXP2
LRRC4B
FUBP3
LRRC4C
CANT1

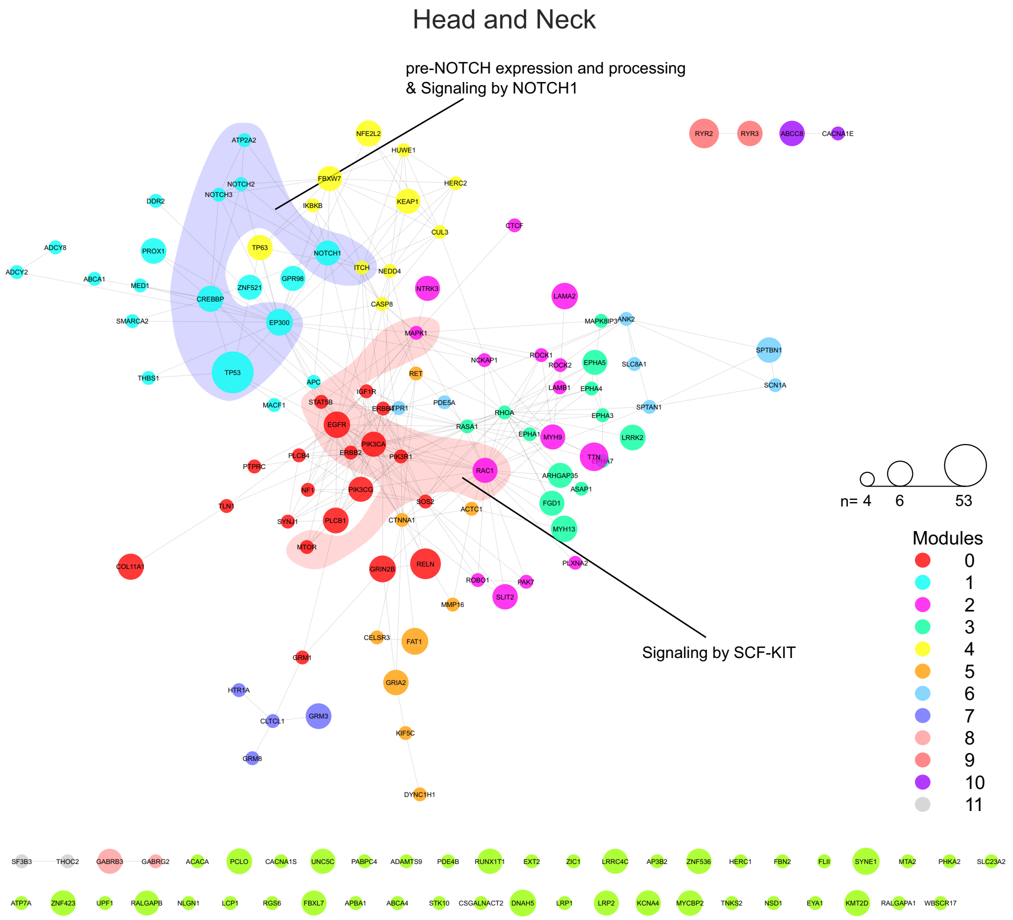
Head and Neck
pre-NOTCH expression and processing
& Signaling by NOTCH1
Signaling by SCF-KIT
n= 4
6
53
Modules
0
1
2
3
4
5
6
7
8
9
10
11
RYR2
RYR3
ABCC8
CACNA1E
NFE2L2
HUWE1
ATP2A2
FBXW7
HERC2
NOTCH2
NOTCH3
KEAP1
DDR2
IKBKB
CUL3
CTCF
ADCY8
PROX1
TP63
NOTCH1
ADCY2
ABCA1
MED1
ZNF521
GPR98
ITCH
NEDD4
NTRK3
CREBBP
CASP8
LAMA2
SMARCA2
EP300
MAPK1
MAPK8IP3
ANK2
SPTBN1
NCKAP1
ROCK1
ROCK2
EPHA5
SLC8A1
TP53
THBS1
APC
RET
LAMB1
EPHA4
SCN1A
IGF1R
MACF1
STAT5B
ERBB4
PTPRT
PDE5A
SPTAN1
RHOA
EPHA3
RASA1
EGFR
PIK3CA
EPHA1
MYH8
LRRK2
PTPRC
PLCB4
ERBB2
PIK3R1
TTN
RAC1
ARHGAP35
NF1
PIK3CG
ASAP1
SOS2
FGD1
ACTC1
TLN1
SYNJ1
PLCB1
CTNNA1
MYH13
MTOR
PLXNA2
COL11A1
RELN
GRIN2B
ROBO1
PAK7
SLIT2
MMP16
CELSR3
FAT1
GRM1
GRIA2
HTR1A
CLTCL1
GRM3
KIF5C
GRM8
DYNC1H1
SF3B3
THOC2
GABRB3
GABRG2
ACACA
PCLO
CACNA1S
UNC5C
PABPC4
ADAMTS9
PDE4B
RUNX1T1
EXT2
ZIC1
LRRC4C
AP3B2
ZNF536
HERC1
FBN2
FLII
SYNE1
MTA2
PHKA2
SLC23A2
ATP7A
ZNF423
UPF1
RALGAPB
NLGN1
LCP1
RGS6
FBXL7
APBA1
ABCA4
STK10
CSGALNACT2
DNAH5
LRP1
LRP2
KCNA4
MYCBP2
TNKS2
NSD1
EYA1
KMT2D
RALGAPA1
WBSCR17

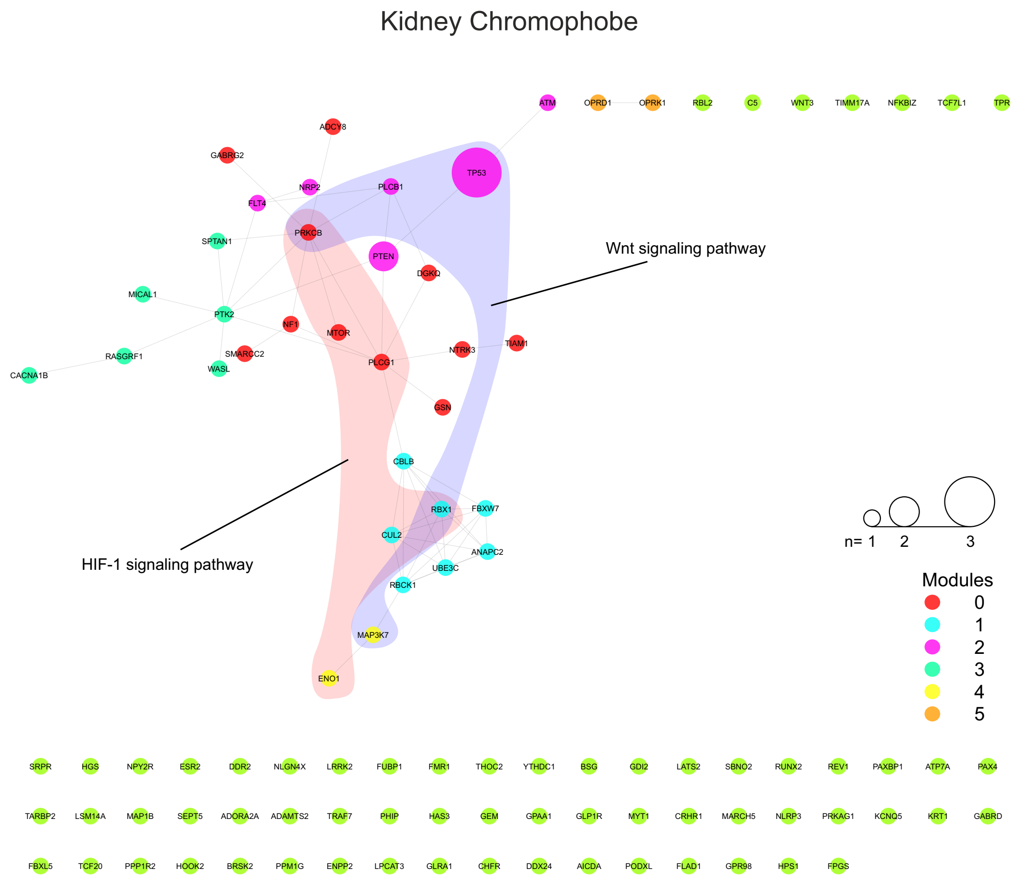

Kidney Chromophobe
Wnt signaling pathway
HIF-1 signaling pathway
ATM
OPRD1
OPRK1
RBL2
C5
WNT3
TIMM17A
NFKBIZ
TCF7L1
TPR
ADCY8
GABRG2
NRP2
PLCB1
TP53
FLT4
SPTAN1
PRKCB
PTEN
DGKQ
MICAL1
PTK2
NF1
MTOR
TIAM1
NTRK3
RASGRF1
SMARCC2
PLCG1
WASL
CACNA1B
GSN
CBLB
RBX1
FBXW7
CUL2
ANAPC2
UBE3C
RBCK1
MAP3K7
ENO1
n= 1
2
3
Modules
0
1
2
3
4
5
SRPR
HGS
NPY2R
ESR2
DDR2
NLGN4X
LRRK2
FUBP1
FMR1
THOC2
YTHDC1
BSG
GDI2
LATS2
SBNO2
RUNX2
REV1
PAXBP1
ATP7A
PAX4
TARBP2
LSM14A
MAP1B
SEPT5
ADORA2A
ADAMTS2
TRAF7
PHIP
HAS3
GEM
GPAA1
GLP1R
MYT1
CRHR1
MARCH5
NLRP3
PRKAG1
KCNQ5
KRT1
GABRD
FBXL5
TCF20
PPP1R2
HOOK2
BRSK2
PPM1G
ENPP2
LPCAT3
GLRA1
CHFR
DDX24
AICDA
PODXL
FLAD1
GPR98
HPS1
FPGS

Kidney Clear Cell

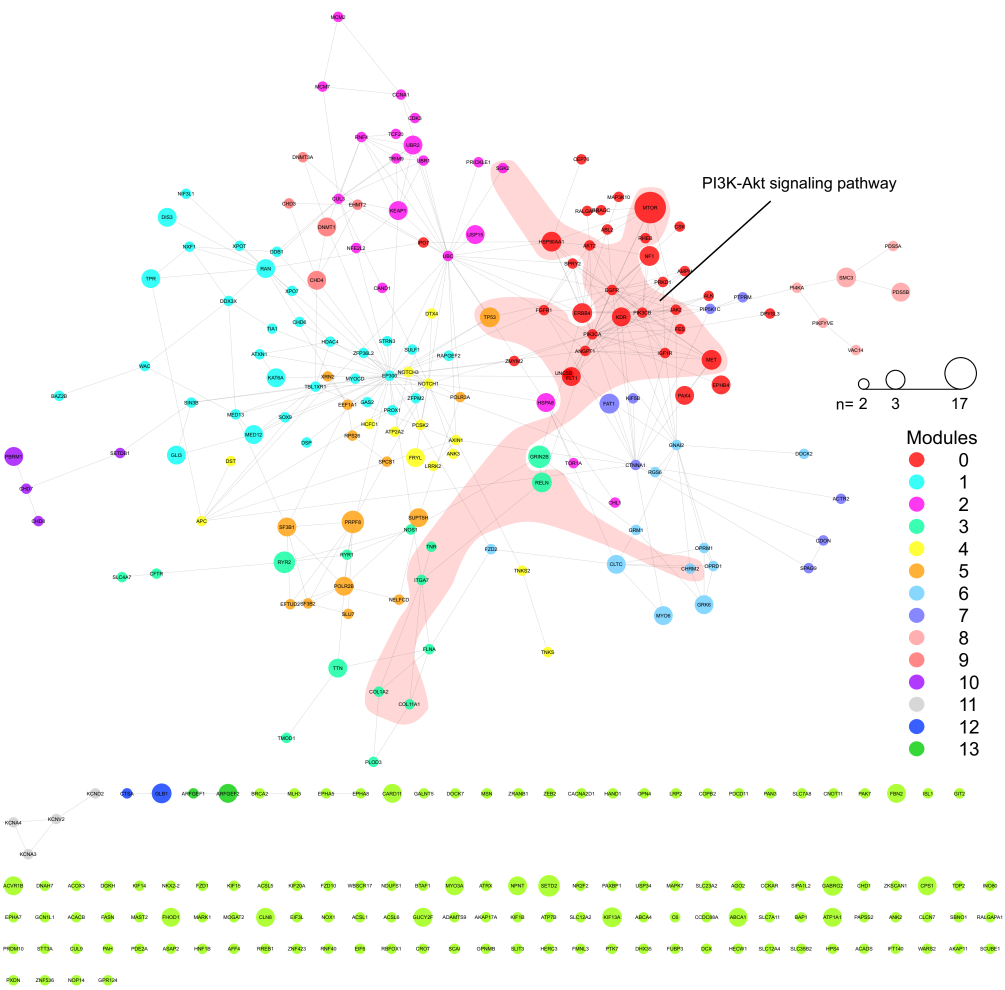

Kidney Papillary

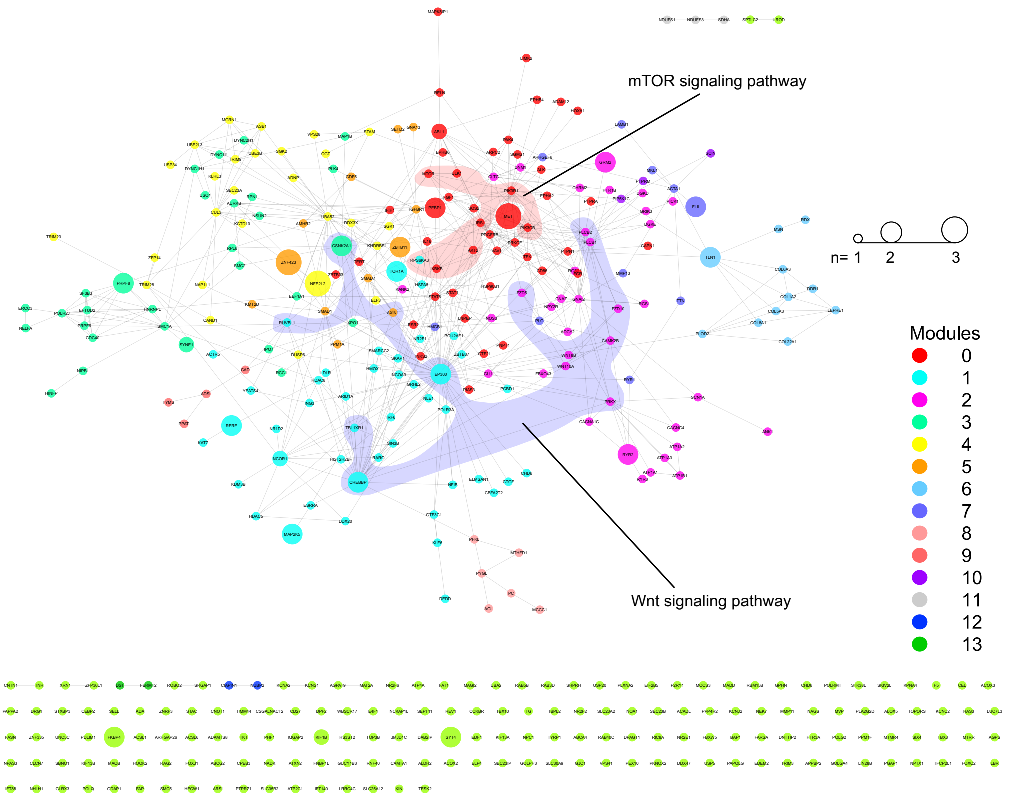

Liver

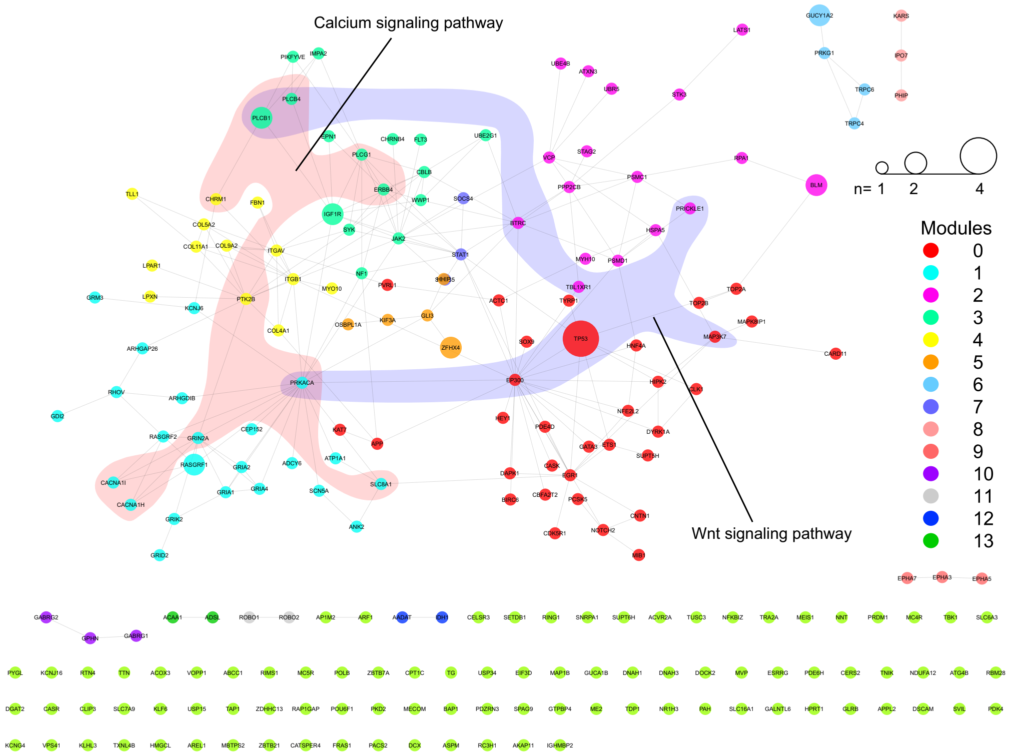

Calcium signaling pathway
Wnt signaling pathway
n= 1
2
4
Modules
0
1
2
3
4
5
6
7
8
9
10
11
12
13

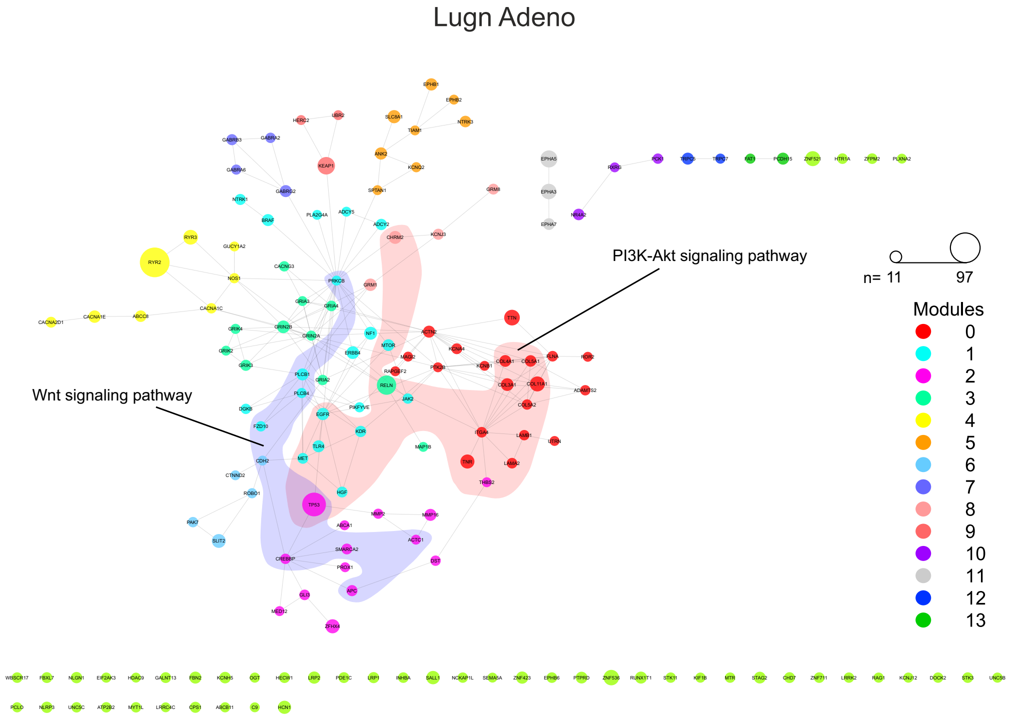

Lugn Adeno
PI3K-Akt signaling pathway
Wnt signaling pathway
n= 11
97
Modules
0
1
2
3
4
5
6
7
8
9
10
11
12
13

Lugn Small Cell

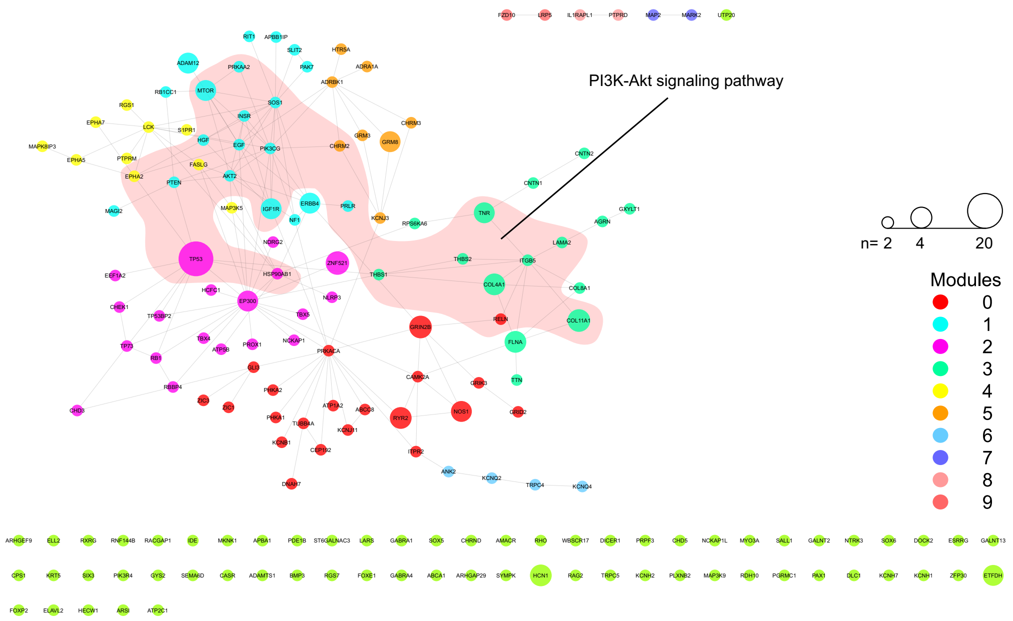

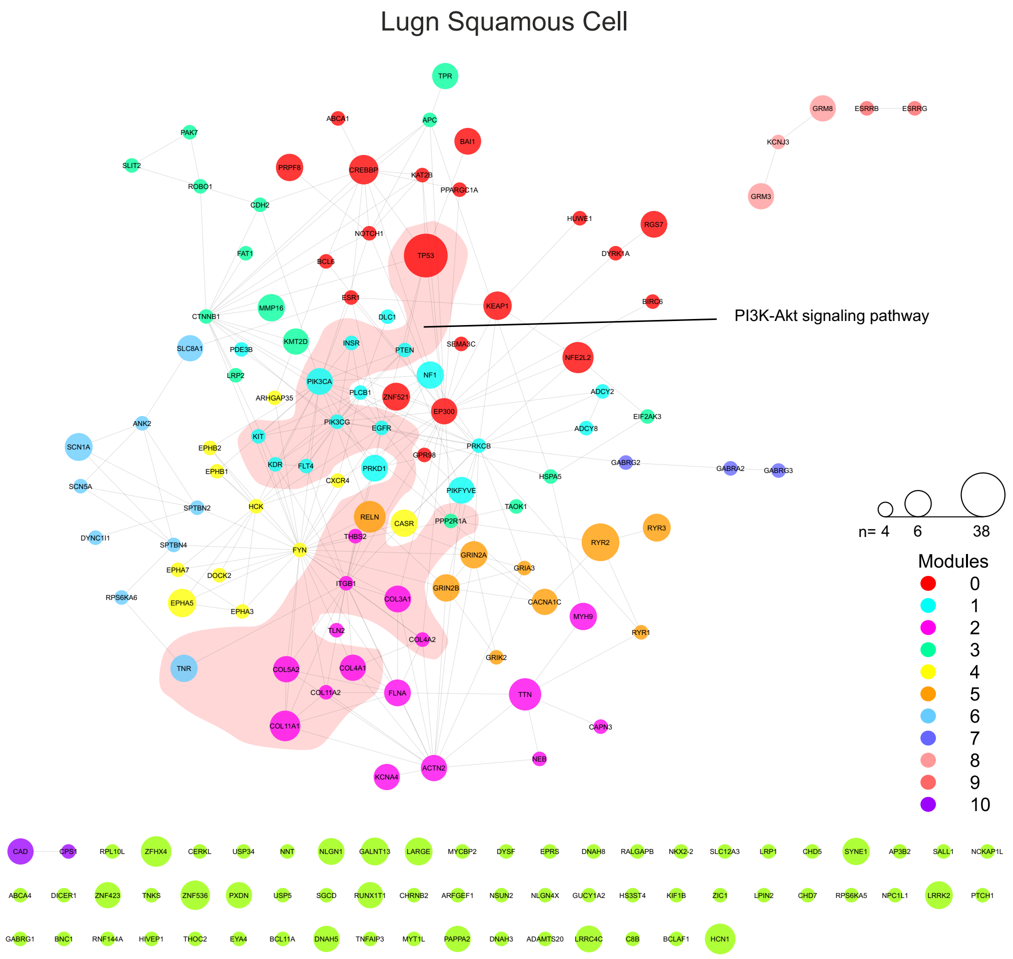
Lugn Squamous Cell
PI3K-Akt signaling pathway
n= 4
6
38
Modules
0
1
2
3
4
5
6
7
8
9
10
TPR
ABCA1
APC
BAI1
PAK7
SLIT2
ROBO1
PRPF8
CREBBP
KAT2B
PPARGC1A
CDH2
HUWE1
RGS7
NOTCH1
TP53
DYRK1A
FAT1
BCL6
ESR1
BIRC6
CTNNB1
MMP16
DLC1
KEAP1
KMT2D
INSR
SLC8A1
PDE3B
PTEN
SEMA3C
NFE2L2
LRP2
NF1
PIK3CA
PLCB1
ZNF521
ADCY2
ARHGAP35
EP300
EIF2AK3
ANK2
PIK3CG
EGFR
ADCY8
SCN1A
KIT
EPHB2
PRKCB
GPR98
GABRG2
GABRA2
GABRG3
KDR
FLT4
PRKD1
EPHB1
SCN5A
CXCR4
HSPA5
PIKFYVE
SPTBN2
HCK
TAOK1
RELN
CASR
PPP2R1A
DYNC1I1
THBS2
RYR3
RYR2
SPTBN4
FYN
GRIN2A
EPHA7
GRIA3
DOCK2
ITGB1
COL3A1
GRIN2B
RPS6KA6
EPHA5
CACNA1C
EPHA3
MYH9
TLN2
RYR1
COL4A2
GRIK2
TNR
COL5A2
COL4A1
COL11A2
FLNA
TTN
COL11A1
CAPN3
NEB
ACTN2
KCNA4
GRM8
ESRRB
ESRRG
KCNJ3
GRM3
CAD
CPS1
RPL10L
ZFHX4
CERKL
USP34
NNT
NLGN1
GALNT13
LARGE
MYCBP2
DYSF
EPRS
DNAH8
RALGAPB
NKX2-2
SLC12A3
LRP1
CHD5
SYNE1
AP3B2
SALL1
NCKAP1L
ABCA4
DICER1
ZNF423
TNKS
ZNF536
PXDN
USP5
SGCD
RUNX1T1
CHRNB2
ARFGEF1
NSUN2
NLGN4X
GUCY1A2
HS3ST4
KIF1B
ZIC1
LPIN2
CHD7
RPS6KA5
NPC1L1
LRRK2
PTCH1
GABRG1
BNC1
RNF144A
HIVEP1
THOC2
EYA4
BCL11A
DNAH5
TNFAIP3
MYT1L
PAPPA2
DNAH3
ADAMTS20
LRRC4C
C8B
BCLAF1
HCN1

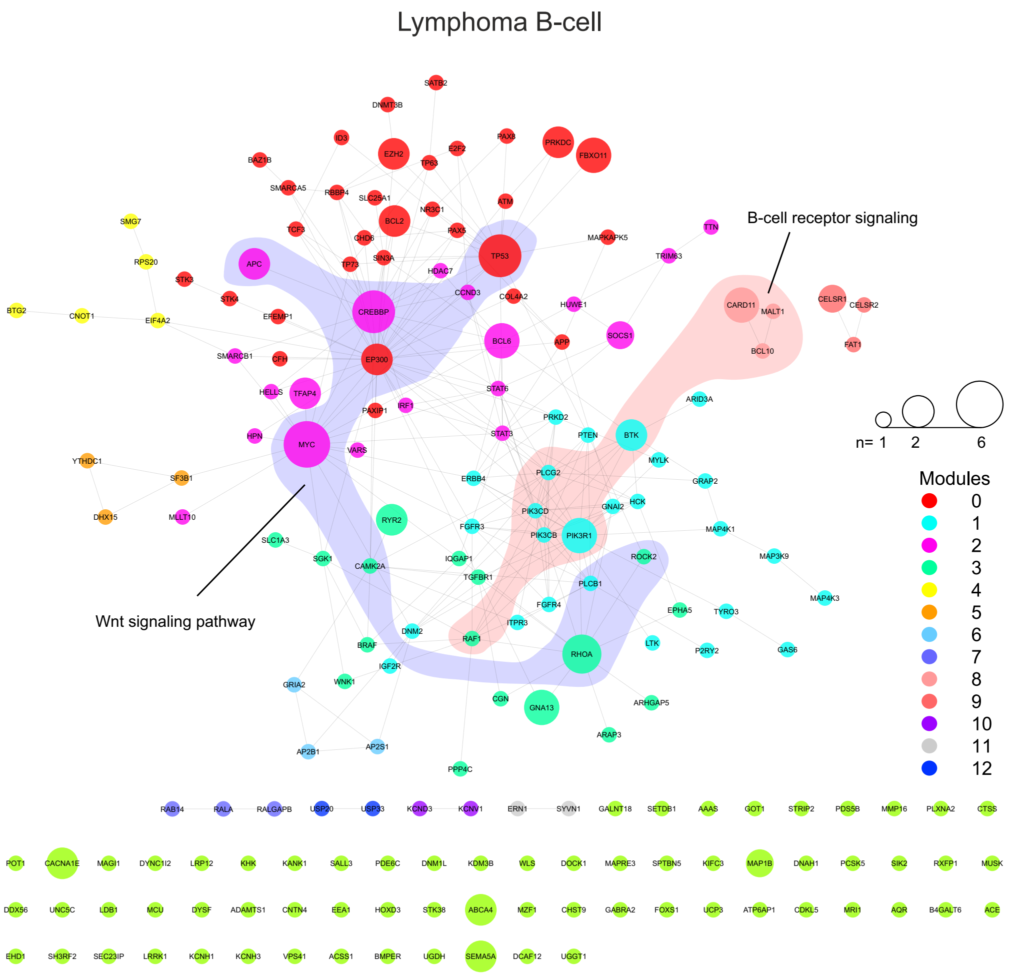

Lymphoma B-cell
B-cell receptor signaling
Wnt signaling pathway
n= 1 2 6
Modules
0
1
2
3
4
5
6
7
8
9
10
11
12
SATB2
DNMT3B
ID3
EZH2
E2F2
PAX8
PRKDC
FBXO11
BAZ1B
TP63
SMARCA5
RBBP4
SLC25A1
NR3C1
ATM
BCL2
TCF3
CHD6
PAX5
SMG7
TTN
MAPKAPK5
RPS20
APC
TP73
SIN3A
HDAC7
TP53
TRIM63
STK3
STK4
CCND3
COL4A2
CARD11
MALT1
CELSR1
CELSR2
BTG2
CNOT1
EIF4A2
EFEMP1
CREBBP
HUWE1
SOCS1
BCL6
APP
BCL10
FAT1
SMARCB1
CFH
EP300
HELLS
TFAP4
STAT6
ARID3A
PAXIP1
IRF1
PRKD2
HPN
MYC
STAT3
PTEN
BTK
VARS
MYLK
YTHDC1
SF3B1
ERBB4
PLCG2
GRAP2
DHX15
MLLT10
RYR2
PIK3CD
GNAI2
HCK
FGFR3
PIK3CB
PIK3R1
MAP4K1
SLC1A3
SGK1
CAMK2A
IQGAP1
ROCK2
MAP3K9
TGFBR1
PLCB1
MAP4K3
FGFR4
EPHA5
TYRO3
DNM2
ITPR3
RAF1
BRAF
LTK
RHOA
P2RY2
GAS6
IGF2R
GRIA2
WNK1
CGN
GNA13
ARHGAP5
ARAP3
AP2B1
AP2S1
PPP4C
RAB14
RALA
RALGAPB
USP20
USP33
KCND3
KCNV1
ERN1
SYVN1
GALNT18
SETDB1
AAAS
GOT1
STRIP2
PDS5B
MMP16
PLXNA2
CTSS
POT1
CACNA1E
MAGI1
DYNC1I2
LRP12
KHK
KANK1
SALL3
PDE6C
DNM1L
KDM3B
WLS
DOCK1
MAPRE3
SPTBN5
KIFC3
MAP1B
DNAH1
PCSK5
SIK2
RXFP1
MUSK
DDX56
UNC5C
LDB1
MCU
DYSF
ADAMTS1
CNTN4
EEA1
HOXD3
STK38
ABCA4
MZF1
CHST9
GABRA2
FOXS1
UCP3
ATP6AP1
CDKL5
MRI1
AQR
B4GALT6
ACE
EHD1
SH3RF2
SEC23IP
LRRK1
KCNH1
KCNH3
VPS41
ACSS1
BMPER
UGDH
SEMA5A
DCAF12
UGGT1

Melanoma

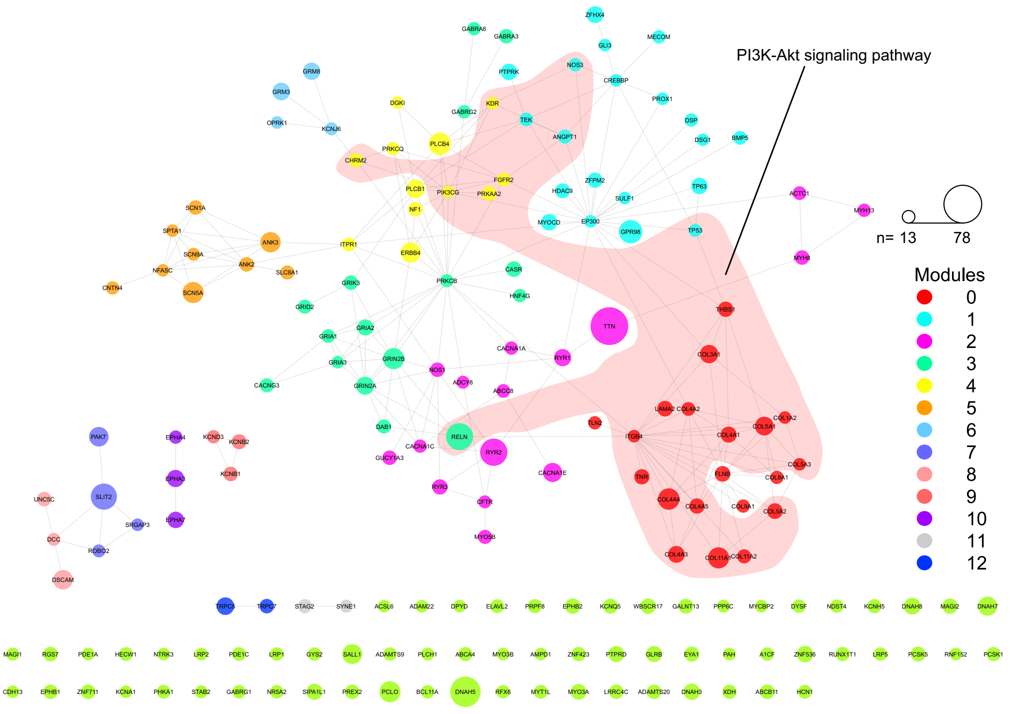

PI3K-Akt signaling pathway
n= 13
78
Modules
0
1
2
3
4
5
6
7
8
9
10
11
12

Myeloma

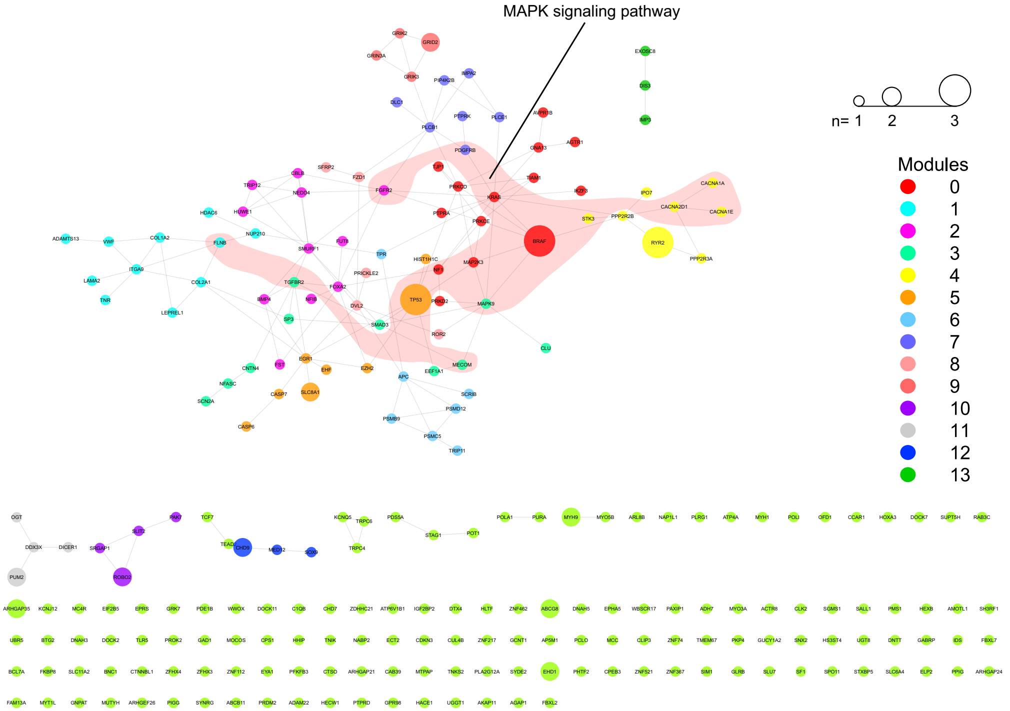

Neuroblastoma

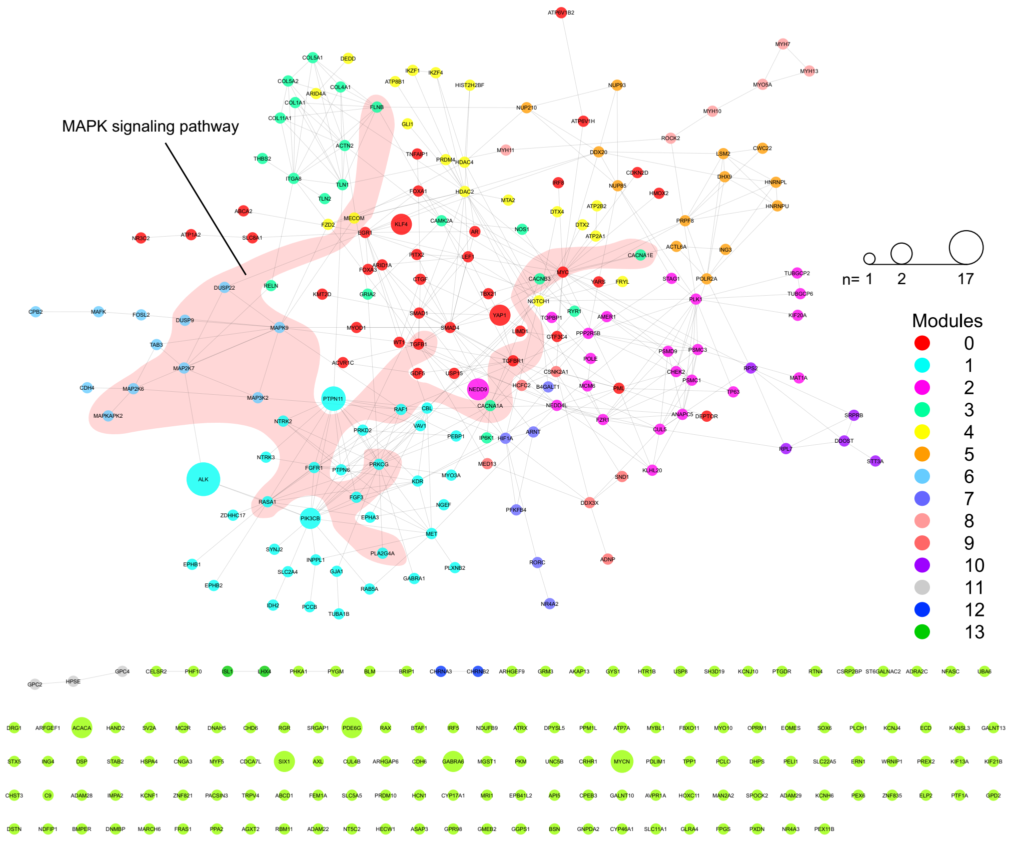

Ovary

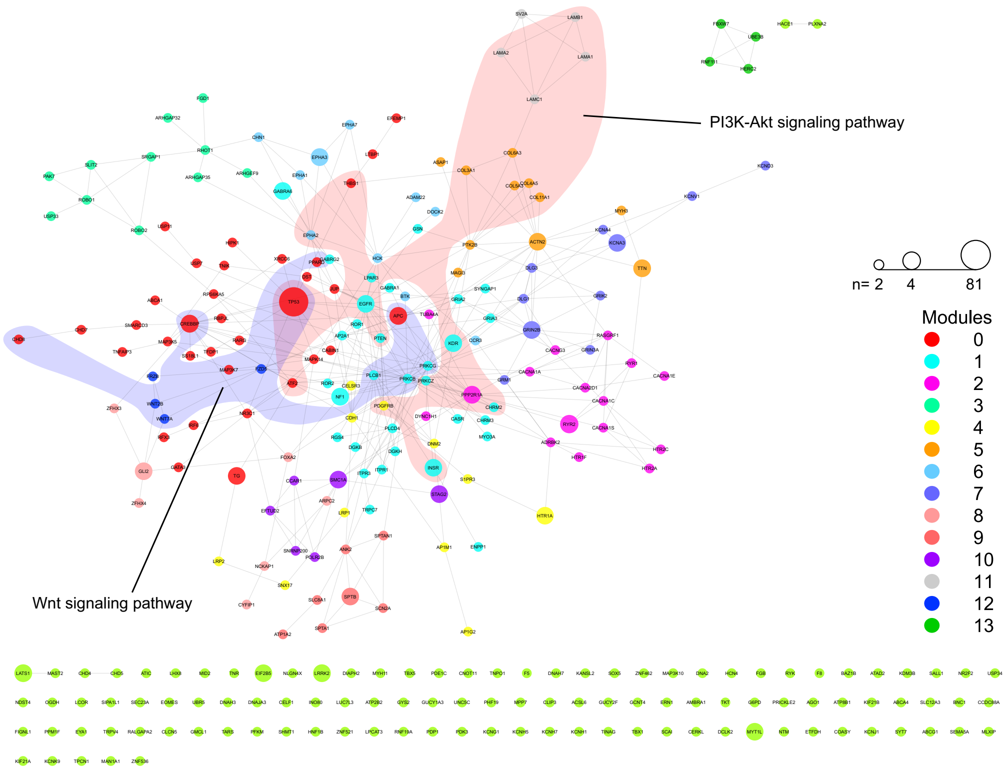
PI3K-Akt signaling pathway
Wnt signaling pathway
n= 2
4
81
Modules
0
1
2
3
4
5
6
7
8
9
10
11
12
13

Pancreas

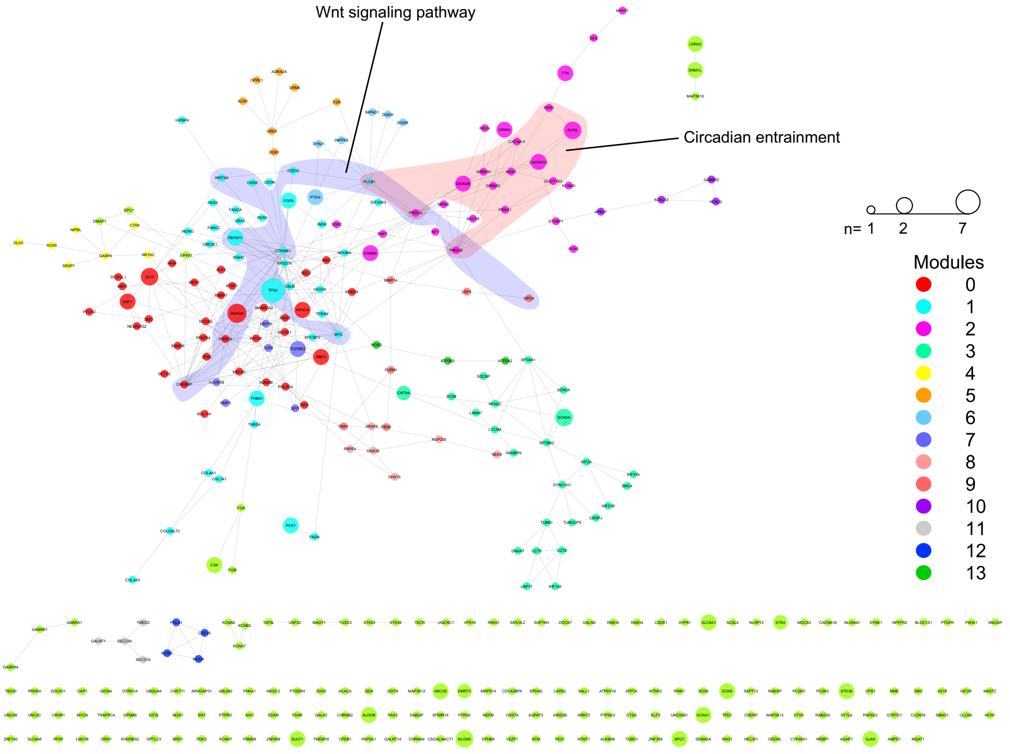

Wnt signaling pathway
Circadian entrainment
n= 1
2
7
Modules
0
1
2
3
4
5
6
7
8
9
10
11
12
13

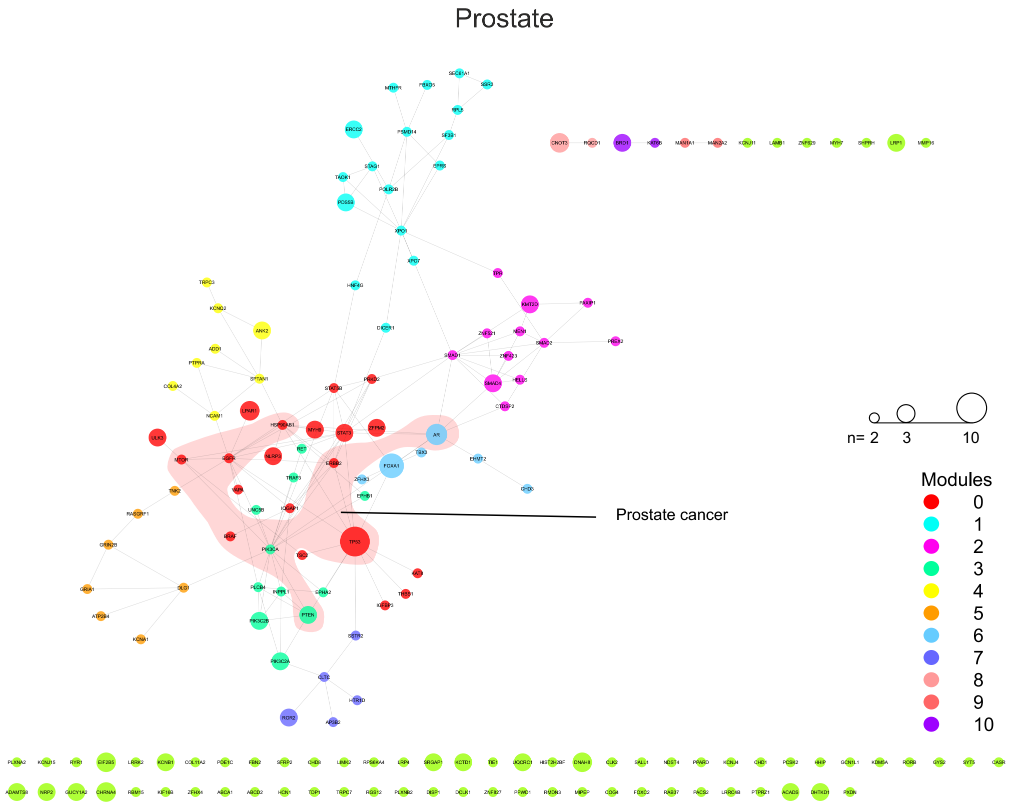
Prostate
Prostate cancer
n= 2
3
10
Modules
0
1
2
3
4
5
6
7
8
9
10

Stomach

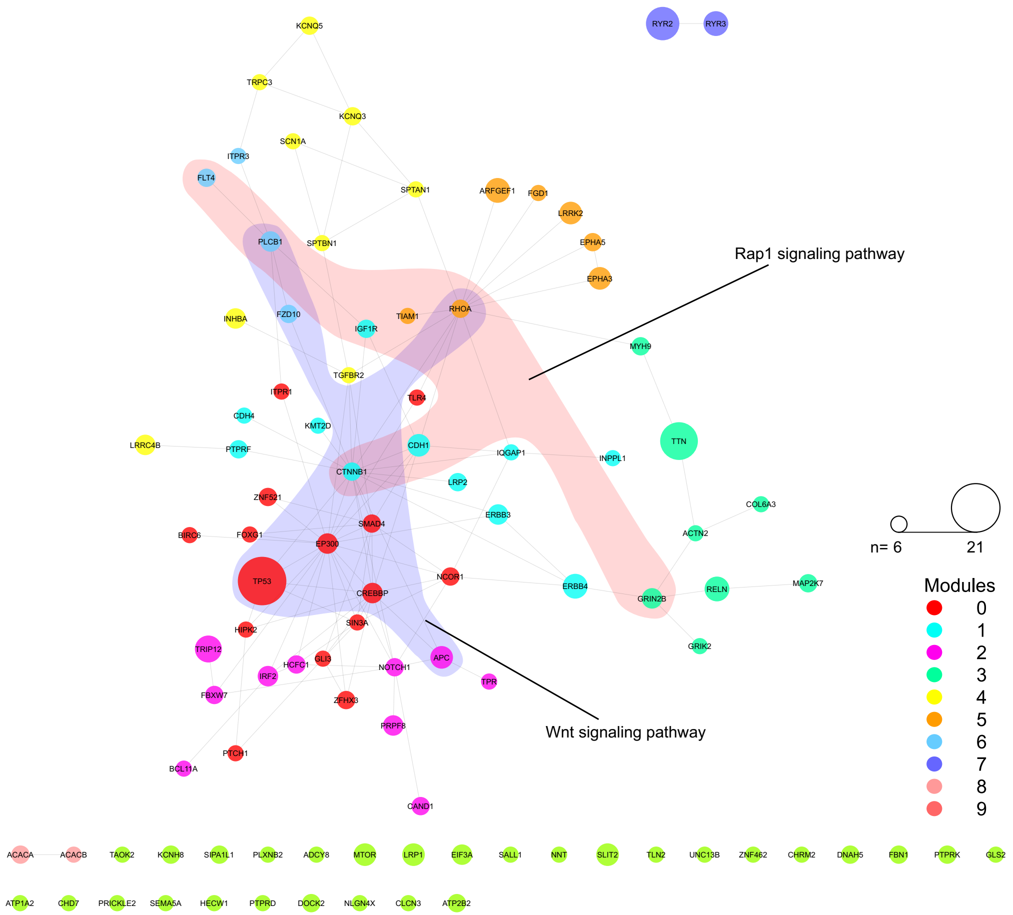
RYR2
RYR3
KCNQ5
TRPC3
KCNQ3
SCN1A
ITPR3
FLT4
SPTAN1
ARFGEF1
FGD1
LRRK2
PLCB1
SPTBN1
EPHA5
EPHA3
Rap1 signaling pathway
INHBA
FZD10
TIAM1
RHOA
IGF1R
MYH9
TGFBR2
ITPR1
TLR4
CDH4
KMT2D
LRRC4B
PTPRF
CDH1
IQGAP1
INPPL1
TTN
CTNNB1
LRP2
ZNF521
COL6A3
ERBB3
SMAD4
BIRC6
FOXG1
EP300
ACTN2
TP53
NCOR1
ERBB4
RELN
MAP2K7
CREBBP
GRIN2B
SIN3A
HIPK2
TRIP12
GRIK2
APC
HCFC1
GLI3
NOTCH1
IRF2
TPR
FBXW7
ZFHX3
PRPF8
Wnt signaling pathway
PTCH1
BCL11A
CAND1
ACACA
ACACB
TAOK2
KCNH8
SIPA1L1
PLXNB2
ADCY8
MTOR
LRP1
EIF3A
SALL1
NNT
SLIT2
TLN2
UNC13B
ZNF462
CHRM2
DNAH5
FBN1
PTPRK
GLS2
ATP1A2
CHD7
PRICKLE2
SEMA5A
HECW1
PTPRD
DOCK2
NLGN4X
CLCN3
ATP2B2
n= 6
21
Modules
0
1
2
3
4
5
6
7
8
9

Thyroid

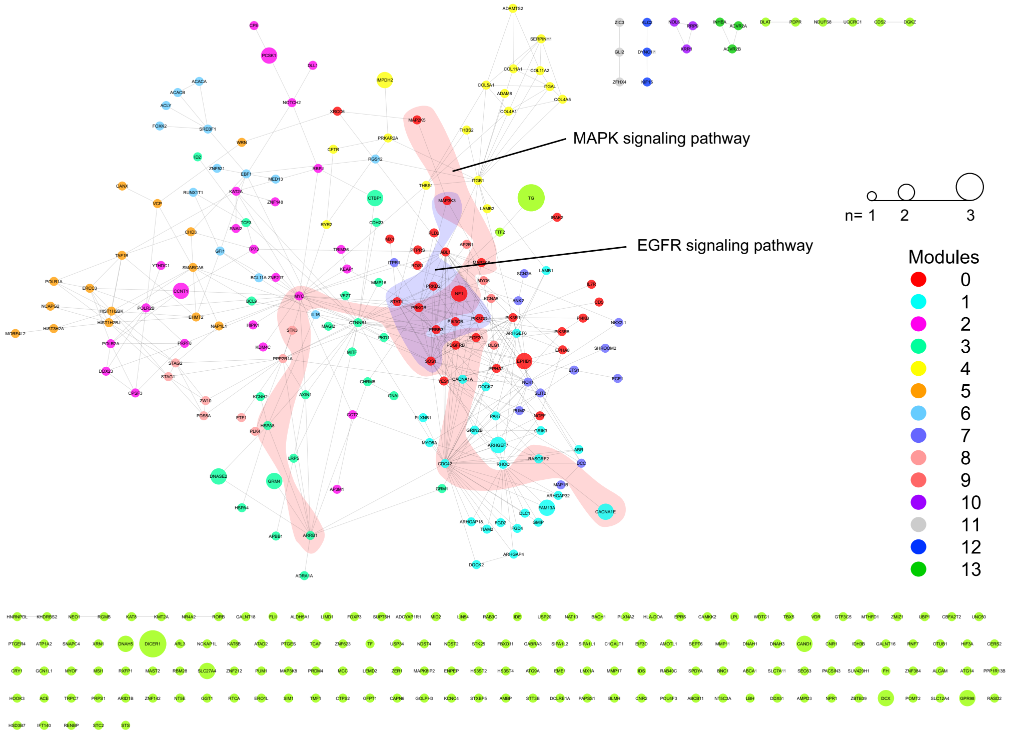

MAPK signaling pathway
EGFR signaling pathway
n= 1 2 3
Modules
0
1
2
3
4
5
6
7
8
9
10
11
12
13

Uterus

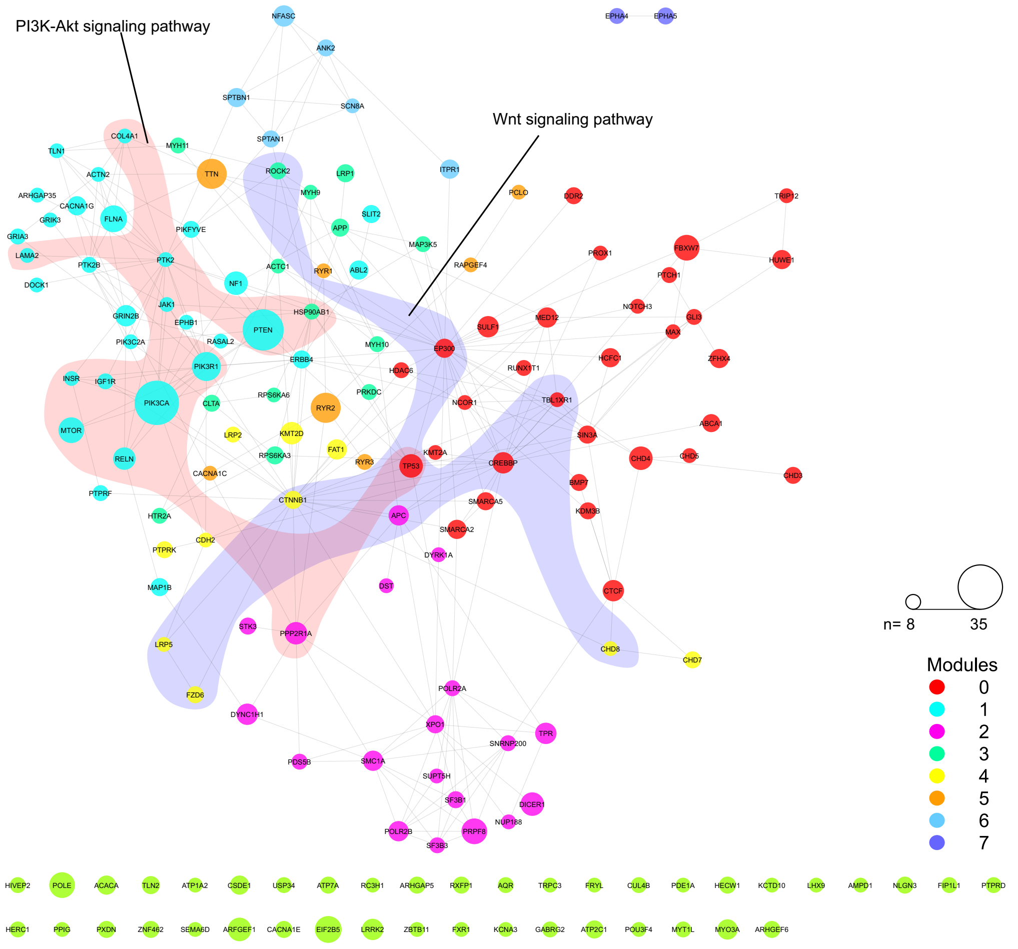
PI3K-Akt signaling pathway
Wnt signaling pathway
n= 8
35
Modules
0
1
2
3
4
5
6
7
